# Computational Analysis of the Homologous Mouse Double RINGs Interface for Targeted Anticancer Drug Design †

**DOI:** 10.3390/biomedicines14102366

**Published:** 2026-10-17

**Authors:** Adeniyi Thompson Adewumi, Lucas Sekele, Wande M. Oluyemi, Shadrach C. Eze, Salerwe Mosebi

**Affiliations:** 1 Department of Life and Consumer Sciences, College of Agriculture and Environmental Sciences, University of South Africa, Roodepoort 1710, South Africa; jesutomisin0707@gmail.com (A.T.A.); sekellst@unisa.ac.za (L.S.); 2 Department of Pharmaceutical and Medicinal Chemistry, College of Pharmacy, Afe Babalola University, Ado-Ekiti 360102, Ekiti State, Nigeria; oluyemiwm@abuad.edu.ng (W.M.O.); shadrachchinecheremeze@gmail.com (S.C.E.)

**Keywords:** cancer surge, target-based anticancer design, MDM2-MDMX RING protein, dynamic structural conformation, binding mechanism, promising inhibitors

## Abstract

**Background/Objectives:** The new yearly 1.1 million cancer cases and considerably increasing mortality, after decades of decline, require urgent and effective therapy. Targeted anticancer therapy is an attractive strategy for treating cancer by targeting critical receptors. The MDM2-MDMX RING heterodimer downregulates the *p53* tumor suppressor pathway, making its disruption a primary therapeutic strategy in oncology. This study evaluates a series of novel inhibitors against the MDM2-MDMX RING interface to identify potent candidates that overcome the binding affinity and structural stability limitations of current small-molecule options like HLI373. **Methods:** Comprehensive computational simulations and MM/PBSA energy calculations were performed on five inhibitor complexes: Hinokiflavone, MMRi62, MMRi64, MMRi71, and HLI373. Structural stability, flexibility, and compactness were rigorously analyzed across a 200 ns molecular dynamics simulation trajectory using Root Mean Square Deviation (RMSD) and Root Mean Square Fluctuation (RMSF) metrics alongside Principal Component Analysis. **Results:** Among the evaluated MDM2-MDMX dual inhibitors, MMRi62 demonstrated the strongest binding affinity with a *ΔG*_bind_ of −26.42 kcal/mol, outperforming reference HLI373 (−14.09 kcal/mol), Hinokiflavone (−22.69 kcal/mol), and MMRi64 (−16.73 kcal/mol). Structurally, MMRi64-bound complexes exhibited the highest stability, achieving the lowest RMSD of 2.28 Å and RMSF of 1.30 Å. Conversely, HLI373-bound complexes displayed pronounced structural fluctuations, yielding a high RMSD of 5.11 Å. **Conclusions:** These inhibitors present highly promising alternatives to HLI373 for targeting the MDM2-MDMX RING interface. MMRi62 and Hinokiflavone, in particular, stand out as highly potent dual-inhibitor leads for future targeted anticancer therapy development.

## 1. Introduction

The absolute numbers of cancer cases and deaths continue to rise globally despite the decrease in age-standardized death rates in many high-income regions due to better treatments and early detection [1,2,3]. Some of the factors contributing to this decline, driven by significant advancements, include precision medicine, immunotherapy, and targeted therapies [4,5]. In 2022, there were an estimated 20 million new cancer cases and 9.7 million deaths globally [6]. Projections indicate a substantial rise to over 30 million new cases and 18 million deaths by 2050 [7]. The most frequently diagnosed cancers worldwide are lung, female breast, and colorectal cancers. Lung cancer remains the leading cause of cancer death, followed by colorectal, liver, and breast cancers [2]. The American Cancer Society estimates 2,041,910 new cancer cases and 618,120 deaths in the United States in 2025 [8]. The overall cancer death rate in the US has been declining since the early 1990s, with a 1.7% annual decrease in men and a 1.3% decrease in women between 2018 and 2022 [9]. More worrisome is the alarming increase in the number of young adults developing and dying from cancer, and presenting with metastatic disease or advanced local disease that is often deadly.

Although cancer therapy is undergoing a rapid transformation towards more personalized, effective, and less invasive treatments, key advancements include precision medicine and targeted therapy (PMTT), immunotherapy, antibody–drug conjugates (ADCs), cancer vaccines, advanced radiation and surgery (ARS), and artificial intelligence (AI). New combination therapies and expanded applications for solid tumors are a major focus of ongoing clinical trials. The recent breakthroughs include FDA approvals for drugs targeting specific mutations, such as BRAF V600E in melanoma and HER2 mutations in breast cancer [10,11].

This work basically elaborates on targeted drug therapy, specifically the criticality of the mouse double minute RING domain homologs (MDM2 RING and MDMX RING) in the design of anticancer drugs. Both RING domains form hetero-oligomers (a stable, elongated complex formed by the C-terminal RING domains of both proteins) that act as a platform for E2 enzyme binding [12]. The C-terminus of MDM2 is often crucial for assembly, structural integrity, and E3 ligase activity [13]. The complex features a central 6-strand *β*-barrel and two C_2_H_2_C_4_ zinc finger motifs [14]. The overexpression of the regulatory mechanism of MDM2-MDMX proteins negatively inactivates the transcriptional activity of the p53 protein [15]. The MDM2 RING domain spans residues 438–491, while the MDMX RING spans residues 437–490, and both mediate critical protein–protein interactions that regulate p53, making this interface a target for cancer therapy [16]. The MDM2-MDMX RING heterodimer is expressed and functions as a critical oncogenic driver in several human cancer types, including breast cancer, soft tissue sarcomas, glioblastomas, retinoblastomas, colorectal cancer, prostate cancer (PC), leukaemia, and lung cancer [17,18]. The MDM2 and MDMX enzymes have also been explored as drug targets in the design of metastatic and triple-negative cancer drugs [18,19]. Figure 1 illustrates the various structural domains of MDM2 and MDMX proteins.

The design of small-molecule inhibitors against the MDM2-MDMX RINGs interface has been a recent highlight of targeted anticancer therapy [20,21]. This interventive approach has been bolstered by the development of Hinokiflavone [HFV (4′, 6″-O-biapigenin)] compared to the quinolinol compounds, “RING-finger inhibitors (*MMRi*)”; *MMRi62* [22], *MMRi64* [23], and *MMRi71* [23]. Hinokiflavone (*Hinok*) directly binds to the MDM2-MDMX RING complex to inhibit its E3 ligase activity and promote the downregulation of MDM2 and MDMX and restore p53 function in cancer cells. [24,25]. Several mechanisms have been reported for the pharmacological activities of *Hinok*, including the inhibition or disruption of MDM2, activation of the mitochondrial ROS/JNK/caspase signaling pathway, metalloproteinase inhibition, and RNA splicing to promote cancer cell apoptosis and growth arrest [24]. The *MMRi* series exerts its therapeutic activities by disrupting the interaction between MDM2 and MDMX (or promoting the degradation of MDMX, leading to p53-independent apoptosis or ferroptosis [22,23]. These compounds are potent disrupters of the MDM2-MDMX RING heterodimer, but the experimental structure determination of the dimer interface has proven challenging [20,26]. Previous studies have reported the mechanisms of action of Hinokiflavone using molecular docking [24]. However, standard rigid or semi-flexible docking algorithms often fail to predict transient binding pockets, treat proteins as rigid structures, ignore solvent effects, and use simplified, non-physical scoring functions, leading to high false-positive (or false-negative) rates and inaccurate binding affinities [27]. Therefore, the mechanisms underlying the effect of the molecules are still unclear. More importantly, the atomistic dynamics of how these inhibitors influence or modulate the transfer of ubiquitin from the E2 conjugating enzyme to substrates have not been completely mapped using classical Molecular Dynamics (MD) simulations. Static docking models fail to capture how proteins change shape. To design an effective inhibitor, the molecule must trap the MDM2-MDMX interface into a stable, inactive state over time. Researchers track this structural transition by measuring key metrics, such as Root Mean Square Deviation to monitor overall structural stability and Root Mean Square Fluctuation to pinpoint local flexibility changes.

Therefore, this study aimed to perform extensive all-atom MD simulations on identified ligand–protein complexes to evaluate the structural stability and rigidity changes induced by ligand binding over a time-dependent simulation trajectory, ensuring that the top-ranked inhibitor suppresses the natural flexibility of the interface and prevents the dynamic shifts required for functional MDM2-MDMX crosstalk. Thus, we evaluate a series of novel inhibitors against the MDM2-MDMX RING interface to identify potent candidates that overcome the binding affinity and structural stability limitations of current small-molecule options like HLI373. This work unravels the underlying dynamic conformational stability of the MDM2-MDMX RING interface and applies advanced free-energy perturbation methods (MMPBSA) to conduct per-residue energy decomposition to isolate critical interaction hotspots that drive heterodimerization. The significance of simulating the MDM2-MDMX interface is justified by the high structural flexibility of the C-terminal RING domains, which have traditionally precluded high-resolution co-crystallization with small-molecule inhibitors.

## 2. Materials and Methods

### 2.1. Retrieval and Preparation of Targets and Inhibitors

The MDM2-MDMX heterodimer, rather than the MDM2 homodimer, is often the target for disrupting E3 ligase activity with small-molecule inhibitors, such as *MMRi64*. Based on the literature search, there is no publicly available PDB file (as of mid-2026) that contains a co-crystallized structure of the MDM2-MDMX RING domain with a known ligand at the interface. Thus, the 3D structures of the MDM2-MDMX RING domain heterodimer were retrieved from the Protein Data Bank (PDB) under the ID code 2VJE (Chains A, B, C, and D) [20]. Chain C (MDM2 RING) residues range from 428 through 491, while Chain D (MDMX RING) residues range from 428–490, as crystallized. However, only Chains C (MDM2 RING) and D (MDMX RING) were used throughout this study.

The Zn^2+^ ions in the RING domains are coordinated by a conserved network of Cysteine (CYM, deprotonated) and Histidine (HID/HIE) residues. All coordinating cysteines were explicitly converted to CYM residues to accurately reflect their negatively charged, deprotonated states. Rather than deriving system-specific QM/MM covalent parameters [28], such as the ZAFF or MCPB.py workflow), a robust Manual Distance and Angle Restraint workflow [29,30] was enforced. This maintains the tetrahedral coordination geometry (T_d_) of the four-to-one sulfur/nitrogen arrangement around each Zn^2+^ center. Harmonic distance restraints are applied between the Zn^2+^ ion and the coordinating heavy atoms: the sulfur atoms (S_γ_) of Cys and the epsilon/delta nitrogens (N_ε_/N_δ_) of His. The equilibrium distance (r_0_) was set to 2.35 Å for Zn^2+^-S_γ_ bonds and 2.05 Å for Zn^2+^-N bonds based on crystal structure averages. A harmonic potential with a force constant (k_r_) of 25.0 kcal/mol·Å^2^ was applied via the DISANG functionality in AMBER. To prevent the collapse of the coordination sphere into a planar geometry, harmonic angle restraints were introduced for all six unique angles flanking the central Zn^2+^ ion (S_γ_–Zn^2+^–S_γ_ or S_γ_–Zn^2+^–N). The equilibrium Angle (θ_0_) was set to the ideal tetrahedral value of 109.5°, and an angular force constant (k_θ_) of 30.0 kcal/mol·rad^2^ was assigned. The restraints are mapped into an NMR-style restraint input file parsed directly by pmemd.cuda. For each of the five inhibitor complexes, a script identifies the residue index of the Zn^2+^ ions and their respective coordinating ligand atoms. Lastly, we used our in-house restraint topology, which reads the defined distance and angle restraint parameters.

The molecular structures of *Hinok*, *HLI373*, *MMRi62*, *MMRi64*, and *MMRi71* were drawn in MarvinSketch and saved in the *mol2* format. Each inhibitor was assessed using the Molegro Molecular Viewer (MMV) Tool [www.molegro.com] [31] to ensure an appropriate display of bond angles and hybridization state. The 2D structures of the inhibitors were converted to 3D structures and optimized in Avogadro-1.2.0 [32,33]. The coordinates of each ligand were further optimized in UCSF ChimeraTool. The ligands were prepared in the same ChimeraTool by using the add-on including Structure Editing/Dock Prep. By default, solvent was removed, hydrogen atoms were added, and Gasteiger charges were added to each ligand using AMBER FF14SBB, and each ligand was saved as a Mol2 file. The charges on each of *Hinok*, *HLI373*, *MMRi62*, *MMRi64*, and *MMRi71* were 0, 2, 0, 0, and 0, respectively. Figure 2a shows the structure of the MDM2-MDMX RING domain, and Figure 2b–e show the chemical structures of Hinokiflavone, *MMRi62*, *MMRi64*, *MMRi71*, and *HLI373* (reference standard).

### 2.2. Active Site Identification and Molecular Docking Calculations

The MDM2-MDMX RING conformation (PDB ID: 2VJE) was chosen for this study to ensure that the results are comparable with experimental results [6,34,35]. Structural analyses show that the C-terminal regions and specific hydrophobic loops of the RING domains are critical for heterodimer formation [20]. The MDM2-MDMX RING heterodimer interface involves three main interaction regions, with the primary one involving the C-terminus of MDMX binding to the second alpha-helix of MDM2 [20]. The MDMX RING C-terminal contains a cleft that is crucial for interacting with the MDM2 RING and can be targeted by small-molecule inhibitors, such as Hinokiflavone and *MMRi* compounds. There are five C-terminal residues each in the MDM2 RING and MDMX RING subunits of the heterodimer, with specific residues particularly important for burying contacts that form the active site. The key active site residues [20] include MDM2 RING’s Pro431, Asn433, Arg444, His452, Thr455, His457, Thr463, Lys467, and Tyr489, and MDMX’s RING residues Gly447, Leu483, Ile485, Val487, Phe488, Ile489, and Ala490.

However, due to the dynamic nature of PPIs, we further validate the binding site (interface) of the MDM2-MDMX RING domain by integrating structural and computational approaches to identify “hotspot” residues that drive dimerization. First, the *ClusPro* protein–protein docking was performed on the active site of the MDM2-MDMX RING heterodimer interface using the 3D structure (2VJE) (https://cluspro.org/signup.php, accessed on 9 May 2026). Chain C (MDM2 RING) was uploaded as the receptor, while Chain D (MDMX RING) was uploaded as the ligand for protein–protein docking calculation. Second, the interactions were viewed using the *PDBSum* open-source tool-v1.0.1, which maps out the hydrogen bonds of MDM2 residues interacting with the MDMX (Figure 3). The interaction types between the MDM2 RING (Chain C) and MDMX RING (Chain D) include hydrogen bonds (blue), non-bonded contacts, such as hydrophobic interactions (brown), salt bridges (red), and disulphide bonds (yellow), which are shown as the key/legend in Figure 3.

Following the protein–protein docking, molecular docking calculations of the inhibitors to the active site of the MDM2-MDMX RING domain were performed, as follows. The MDM2-MDMX RING domain and the ligands were prepared using UCSF Chimera-1.17.3 and further processed with AutoDock Tools 1.5.6 to convert the mol2 files to PDBQT [36]. The standard docking calculations were performed on Cygwin terminal-3.5.0, which provides a Unix-like environment on Windows to enable the running of Unix commands and applications. The grid box was generated with the AutoDock Tool-1.5.6 was set to the X, Y, Z coordinates: centers 51.849, 37.15, 12.569, and sizes 48, 40, 44, respectively. Docking each ligand to the active site of the MDM2-MDMX RING complex was performed using AutoDock Vina [37] on the Cygwin bash shell open-source terminal [38]. The residue–ligand interactions and their positions, along with bond distances and angles, were visualized in BIOVIA Discovery Studio Visualizer [39].

Lastly, we performed blind docking studies using the Mega-Box Strategy (Standard Vina). The macromolecule grid tool was opened after loading the prepared receptor into the working space. The box size coordinates were stretched to explicitly encapsulate the entire geometric volume of both RING proteins. The grid box obtained for blind docking was set to the X, Y, Z coordinates: sizes 48.591, 22.078, 13.03, and dimensions 112, 120, 108, respectively. For this reason, we chose to increase the exhaustiveness of the docking to 32 and 20 modes, as against 8 that were used in the standard docking where the active site of the protein was specified.

### 2.3. Molecular Dynamics (MD) Simulations

The docked complexes were used to run the MD simulation study, with each ligand separated from its respective MDM2-MDMX RING complex and prepared. In addition, an unbound form of the receptor was also prepared for simulations. The systems were prepared for 200 ns MD simulations using our previously reported protocols [40]. The MD simulations were run using the Graphics Processing Unit (GPU) version of PMEMD.CUDA engine was furnished with the AMBER18 package, using the FF18SB variant force field, and coupled with integrated modules [41]. The Graphics Processing Unit (GPU) version of the AMBER18 software (Particle Mesh Ewald Molecular Dynamics, PMEMD), coupled with its integrated modules, was used to perform MD simulations [41] and to compute long-range electrostatic interactions. ANTECHAMBER was used to parameterize the respective ligands, while the ligand–receptor complexes were optimized with the LEAP module, which also generated respective topologies and parameter files [40,42]. The modification, renaming, and protonation (histidine) of the MDM2-MDMX RING heterodimer protein were achieved using the in-house *pdb4amber* script [41]. At the same time, the topology and parameter files for the respective ligand-bound complexes were created using the LEAP module [41].

Moreover, the module was used to neutralize the systems by adding counter ions (Na^+^ or Cl^−^ ions) at a constant pH with solvation within a TIP3P water box of 12 Å [43]. The system was partially minimized for 5000 steps using a 500 kcal/mol Å^2^ restraint potential, and the full minimization of 10000 steps was performed with an energy restraint. The heating stage was carried out by gradually elevating the system temperature from 0 to 300 K in a canonical ensemble (NVT) for 50 ps using a Langevin thermostat and a 5 kcal/mol Å [44]. Furthermore, the system equilibration was run for 1 ns (50,0000 steps) at 300 K without restraints, while maintaining 1 bar pressure with a Berendsen barostat [45]. The MD production stage of a 200 ns duplicate was performed, where all the atomic hydrogen bonds were constrained, which prevents unphysical artifacts and allows researchers to use a larger time-step [46,47]. The resulting coordinates and trajectories were saved at every time-step of 1 ps and analyzed with the integrated CPPTRAJ and PTRAJ modules [48]. The geometric restraints file was actively called in the &cntrl section using nmropt=1.

The whole steps from minimization to production MD were repeated for every system. Moreover, a second 200 ns (200,000 frames) simulation was repeated (duplicated) for bound and unbound systems. To treat the 200 ns duplicates (replicas) of a simulation trajectory for post-MD analysis, we leveraged the combined trajectories to boost statistical sampling and assess reproducibility rather than simply picking the “best” loop. First, the trajectories were analyzed independently by running structural calculations separately for all replica files. Then, a statistical variation method was used to determine if the observed data were reproducible or occurred by random chance, which allows independent analysis of each 200 ns by computing the mean and standard error of replicas 1 and 2. The output data files were compiled with a .dat extension to be processed. Moreover, nine (9) snapshots were generated at different times/frames (from 0 to 200 ns), which include 1 ns (1000 frames), 25 ns (25,000 frames), 50 ns, 75 ns, 100 ns, 125 ns, 150 ns, 175 ns, and 200 ns.

Lastly, the data plots, mean values, and standard deviations/error across the replicates were obtained with the Origin 6 analytical software [49], while the Graphical User Interface (GUI) of the BIOVIA Discovery Studio Client 2024 and UCSF Chimera-1.17.3 were used for system visualization. Lastly, post-production molecular dynamics simulations were conducted by computing some metrics, including root mean square deviation (RMSD), root mean square fluctuation (RMSF), radius of gyration (RoG), principal component analysis (PCA), Dynamic Cross-Correlation Matrix (DCCM), Define Secondary Structure of Proteins (DSSP), and distances among critical active site residues [50,51].

### 2.4. Post-MD Simulation Analysis

#### 2.4.1. Root Mean Square Deviation (RMSD) and Fluctuation (RMSF)

Root mean square deviation (RMSD) and root mean square fluctuation (RMSF) are fundamental post-simulation analyses in molecular dynamics (MD) simulation studies, acting as critical metrics for validating structural stability and flexibility, respectively [52,53]. They reveal how proteins hold their shape, how ligands bind, and which amino acids contribute most to functional motion.

$${RMSD}_{\left(t\right)}\;=\;\sqrt{\frac{1}{N}\;}\sum_{i=1}^{N}{\left|r_{i}\left(t\right)\;-\;r_{i}^{ref}\right|}^{2}$$

N = Total number of atoms. In most protein simulations, this refers specifically to the number of atoms being compared (often just Cα or backbone atoms). Here, N = the total number of C_α_ atoms. t = Time; this indicates that the RMSD is calculated at a specific time-step (*t*) during a molecular dynamics simulation; = Reference structure; this represents the “gold standard” or starting structure used for comparison, typically the initial experimental crystal structure. Lastly, *r* = Position vector; $r_{i}\left(t\right)$ represents the Cartesian coordinates (x, y, z) of atom i at time t while $r_{i}^{ref}$ represents the coordinates of the same atom in the reference structure.

RMSD measures the average distance between a group of atoms (usually the protein backbone (*C_α_*) of a structure relative to a reference structure (typically the starting crystal structure) at a given time [54]. This metric provides a global measure of conformational change over the entire simulation time. A stable, plateauing RMSD plot indicates that the protein has equilibrated, meaning it is no longer undergoing massive conformational changes from the starting structure. Low RMSD values (~1–3 Å) suggest a stable simulation and remain close to the reference, while high or continuously rising values suggest significant unfolding or conformational shifts. In complex simulations, RMSD is calculated for the protein and ligand separately to verify if the ligand remains bound and stable within the binding site. Comparing RMSD curves allows researchers to identify which protein system is more stable.

On the other hand, RMSF measures the average deviation of a specific particle (residue or atom) from its mean position over the entire trajectory. Unlike RMSD (time-dependent), RMSF is time-averaged, providing a residue-wise picture of local flexibility. High RMSF values identify flexible regions (e.g., loops), while low values indicate rigid regions (e.g., α-helices or β-sheets). It allows identifying key residues in the active site that contribute to ligand binding or induced-fit mechanisms. RMSF is used to assess how a specific ligand binding rigidifies or increases the flexibility of a protein’s structure. It helps determine if ligand binding stabilizes specific parts of the protein compared to the apo form (unbound).

$${RMSF}_{i}\;=\;\sqrt{\frac{1}{T}\sum_{t=1}^{T}{\left|r_{i}\left(t\right)\;-\;{\bar{r}}_{i}\right|}^{2}}$$

Variable explained as i: the specific *residue number* (*or atom*) being analyzed, T: the Total *time* (number of frames) in the simulation, *r_i_*(*t*): the *position* of residue i at a specific time t, and ${\bar{r}}_{i}$: the *average position* of the residue i over the entire simulation.

#### 2.4.2. Radius of Gyration (RoG)and Principal Component Analysis (PCA)

Radius of Gyration (RoG) [55] and Principal Component Analysis (PCA) [56] are indispensable post-simulation analytical tools in Molecular Dynamics (MD) studies, providing insights into the structural compactness and essential motions of protein–ligand complexes, respectively. While RoG gives an overall, scalar measure of folding, PCA acts as a dimensionality reduction technique to visualize complex, collective conformational changes. The radius of gyration measures the compactness of a protein by calculating the root-mean-square distance of its atoms from their common center of mass (CoM). A lower RoG value indicates a more compact, folded structure, whereas a higher value suggests an expanded or unfolded state. RoG is plotted over time to check if the protein structure remains stable or undergoes unfolding/expansion during the simulation. In ligand binding studies, a decrease in the RoG of the protein compared to the apo protein (unbound) indicates that the ligand induces a more stable, compact structure. Sudden jumps in RoG can identify structural transformations, such as the melting of a protein or opening of a domain.

PCA reduces the dimensionality of complex MD trajectory data to identify the essential dynamics, the few dominant motions that account for most of the conformational variance. It constructs a covariance matrix of atomic movements (*C_α_* or backbone atoms) and diagonalizes it to produce eigenvectors (directions of motion) and eigenvalues (magnitude of motion). PCA plots (PC1 vs. PC2) show how the protein explores different conformations. Stable states appear as distinct clusters on these plots. PCA allows researchers to ignore random thermal fluctuations (small eigenvalues) and focus on large-scale collective motions (large eigenvalues) that are biologically relevant. PCA is used to compare the conformational space occupied by the protein–ligand complex with that of the apo protein. A more confined conformational space in the complex indicates stable ligand binding. Porcupine plots, derived from the first few principal components, are used to visualize the direction and amplitude of the most significant structural changes.

#### 2.4.3. Binding Free Energy Calculations

Molecular Mechanics/Poisson–Boltzmann Surface Area (MM/PBSA) method is a popular post-processing method or endpoint energy calculation used to estimate the binding free energy of a ligand to a receptor (such as a protein) from molecular dynamics (MD) simulations [57]. The differential binding of Hinokiflavone, *MMRi62*, *MMRi64*, and *MMRi71* to the MDM2-MDMX RING complex was estimated using MM/PBSA [58]. Mathematically, the binding free energy (ΔGbind) is calculated as the difference in free energy between the complex and the isolated receptor and ligand:

(1) *ΔG_bind_* = *G_complex_* − *G_protein_* − *G_inhibitor_*

For each state (complex, receptor, and ligand), the free energy *G* is broken down into three major terms:

(2) *ΔG_bind_* = *E_gas_* + *G_sol_* − *TS* (or *ΔG_bind_* = *E_MM_* + *G_sol_* − *TS*)

Equation (2) computes the net binding affinity (*ΔG*) between a receptor and a ligand, which is obtained by subtracting the individual components given in Equation (1). *E_gas_* (often representing Δ*E_MM_*) is the gas-phase energy change upon binding; *E_int_* is the internal energy Molecular Mechanics Energy, *E_MM_*, was calculated using Amber standard molecular mechanics force fields, and it includes internal bonded energies (bonds, angles, dihedrals) and non-bonded interactions (electrostatic and van der Waals forces) in a vacuum:

(3) E_gas_ = E_int_ + E_vdw_ + E_ele_

(4) G_sol_ = G_GB_ + G_SA_

(5) G_SA_ = γSASA

To carry out the MM/PBSA calculation, the first 40,000 frames (40 ns) of the first trajectory (200 ns MD simulations) were used by specifying the “startframe” to be equal to 1000 and “endframe” equal to 40,000 at an interval equal to 50 in the mmpbsa input file used to run the post-production MD analysis.

*E_ele_* and *E_vdw_* are the Coulomb and van der Waals energies, respectively. Also, *G_sol_* is the solvation free energy, and *G_GB_* is the polar solvation, which was calculated by solving the Poisson–Boltzmann (PB) equation to evaluate electrostatic interactions between the solute and the surrounding polar solvent. As shown in Equation (5), *G_SA_* is the non-polar solvation contribution and was estimated using an empirical relation proportional to the solvent-accessible surface area (SASA) determined with a water probe radius of 1.4 Å, to capture hydrophobic effects. The surface tension constant *γ* was set to 0.0072 kcal/(mol·Å^2^). The entropy term S (often represented as TΔS) is the total change in entropy resulting from the binding process, and represents the change in rotational, translational, and vibrational degrees of freedom upon binding. The TΔS was ignored in this study due to high computational cost and large statistical noise. T is the temperature in kelvin (° K).

#### 2.4.4. Dynamic Cross-Correlation Matrix (DCCM)

This metric is used graphically to represent the dynamical motions of constituent protein residues over time [51]. Thus, we investigated the inter-residue dynamics and motions of the MDM2-MDMX RING when bound to *Hinok*, *MMRi62*, *MMRi64*, and *MMRi71*, with respect to their inhibition of the E3 ubiquitin ligase activity of the MDM2-MDMX complex, which leads to p53-dependent and independent apoptosis. We obtained the DCCM to estimate the cross-correlated displacement of protein backbone atoms from the resulting trajectories across the MD simulation time according to the equation below:

*Cij* = <∆*ri* ∗ ∆*rj*>/(<∆*r2i*> <∆*r2j*>) 1/2

where *Δri* and *Δrj* depict the mean displacement of the *ith* and *jth* atoms, which are represented by *i* and *dd*, respectively. *Cij* is the coefficient of cross-correlation, which varies from −1 to + 1, corresponding to strongly correlated (+) and anti-correlated (−) motions of constituent residues during the period of MD simulation. This analysis was performed using the integrated CPPTRAJ module [48] of AMBER20 GPU, and the resulting matrices were plotted using Origin data analysis software [49].

#### 2.4.5. Define Secondary Structure of Protein (DSSP)

The Define Secondary Structure of Proteins (DSSP) algorithm is the standard tool for assigning secondary structure components, such as α-helices, β-sheets, and loops, to amino acid residues based on protein atomic coordinates [59]. In Molecular Dynamics (MD) simulations, DSSP is critical for translating the vast amount of raw atomic position data into a clear, time-dependent map of how a protein’s structure evolves. DSSP determines secondary structure primarily by analyzing the hydrogen bonding pattern of the protein backbone, rather than relying solely on dihedral angles (*ϕ*, *ψ*). It calculates the electrostatic energy between all donor–acceptor pairs, identifying a hydrogen bond if the energy falls below a specific threshold (*E* < −0.5 kcal/mol). DSSP defines eight (8) distinct structural states based on H-bond patterns (3-10 helix, α-helix, π-helix, turn, bend, β-sheet, β-bridge, and coil). When applied to MD simulations, DSSP analyzes every frame of the trajectory, enabling researchers to track the formation and disruption of secondary structures over time. It requires high-resolution 3D atomic coordinates (e.g., from PDB files or MD simulation frames), typically utilizing nitrogen, carbon, alpha-carbon, and oxygen atoms.

## 3. Results

### 3.1. Molecular Docking Analysis

Table 1 shows the binding affinities of the ligands binding to the MDM2-MDMX RING heterodimer interface obtained from the standard and blind docking calculations and their respective IC50 values, for comparative and correlation analyses. 

**Table 1:** Correlation of molecular docking, blind docking, and cross-docking results with the reported IC50 of inhibitor-MDM2-MDMX RING interface.

Complex	Standard Docking Score (kcal/mol)	Blind Docking Score (kcal/mol)	IC50 (μM)
*Hinok*-bound	−7.2 (1st)	−8.2 (1st)	≈13 μM (4th) [24]
*MMRi62*-bound	−5.8 (4th)	−6.3 (3rd)	≈0.12–0.33 μM (1st) [22,23]
*MMRi64*-bound	−6.5 (2nd)	−6.5 (2nd)	–< 0.5–3.0 μM (3rd) [60]
*MMRi71*-bound	−5.9 (3rd)	−5.6 (4th)	0.23–0.29 (2nd) μM [23]
HL373-bound	−4.8 (5th)	−5.0 (5th)	15 μM (5th) [24]

The ClusPro protein-protein docking analysis identified the MDM2 RING residues that interacted with the MDMX RING residues at the interface of the heterodimer. Table 2 shows the residues and respective atom that interacted from both RING structure.

**Table 2:** Residues and respective atoms that formed hydrogen bonds at the MDM2 RING and MDMX RING interface (active site), using *ClusPro*-2.0 protein–protein docking software (https://cluspro.org/signup.php) to validate the active site.

MDM2 RING (Chain C)		MDMX RING (Chain D)	
Residue	Atom Name	Residue	Atom Name
Lys446	NZ	Ala490	O
Lys454	O	Lys486	N
Gly456	N	Lys486	O
Gly456	O	Phe488	N
Leu458	N	Phe488	O
Leu458	O	Ala490	N
Leu487	N	Arg453	O
Leu487	O	Gly455	N
Tyr489	N	Gly455	O
Tyr489	O	Leu457	N

### 3.2. Post−MD Simulations Analysis

#### 3.2.1. Binding Interaction Networks of Ligand-Bound MDM2-MDMX RING Complexes

Figure 4, Figure 5, Figure 6, Figure 7 and Figure 8 illustrate the structural binding profiles, 2D interaction networks, and per-residue binding energy contributions of the four selected chemical compounds (L1/Hinok., L2/*MMRi62*, L3/*MMRi64*, L4/*MMRi71*, and L5/*HLI373*) complexed with the MDM2-MDMX RING heterodimer interface. For the molecular dynamics simulations and 3D Conformational Visuals, Figure 4a displays the surface visual of L1/Hinok. (cornflower green) complexed with MDM2-MDMX. For the 3D Inter-Residue Interaction Networks, the corresponding images (b) in Figure 4, Figure 5, Figure 6 and Figure 7 characterize the exact chemical forces governing the complex stability: L1/Hinok. (Figure 4b) is stabilized primarily by a conventional hydrogen bond (Cys441), carbon–hydrogen bonds (Cys441, Arg444), pi–cation forces, pi–alkyl interactions, and pi–sulfur interactions with key residues like Cys441, Arg444, Cys475, Pro474, Asn447, and Leu468. Figure 4c (L1/Hinok.) reveals that Cys441, Arg444, Asn447, Leu468, Cys404, and Leu467 act as vital drivers, with van der Waals and total energy contribution profiles showing major stability hits below −3.5 kcal/mol.

Figure 5a depicts the surface visual of L2/*MMRi62* (dark cyan) complexed with MDM2-MDMX (orange). (Figure 5b) is governed by an extended network including conventional hydrogen bonds, carbon–hydrogen bonds, amide pi-stacked forces, alkyl/pi–alkyl groups, pi–sigma interactions, and pi–cation interactions involving Cys475, Pro474, Ile450, Lys473, Leu468, and Pro476. Figure 5c (L2/*MMRi62*) demonstrates stabilizing energy contributions spanning residues Met459, Leu468, Ala460, Lys473, Pro474, Cys475, and Pro476.

Figure 6a shows the surface visual of L3/*MMRi64* (goldenrod) complexed with MDM2-MDMX (magenta). L3/*MMRi64* (Figure 6b) is anchored by carbon–hydrogen bonds, alkyl hydrophobic forces, pi–alkyl hydrophobic networks, pi–cation interactions, and sulfur interactions across Pro476, Lys473, Leu468, Lys467, Cys404, Ala460, and Met459. Figure 6c (L3/*MMRi64*) highlights high electrostatic and total energy spikes, specifically driven by residues Lys473, Lys467, and Met459.

Figure 7a represents the surface visual of L4/*MMRi71* (orchid) complexed with MDM2-MDMX (olive drab) protein. L2/*MMRi62*L4/*MMRi71* (Figure 7b) is maintained through conventional hydrogen bonds, carbon–hydrogen bonds, and pi–alkyl groupings interacting directly with Pro445, Arg444, Pro437, and Asn433. For per-residue binding energy contributions, images (c) break down the cumulative binding energy contributions (kcal/mol) decomposed into total energy (blue bars), electrostatic energy (green bars), and van der Waals interactions (red bars): Figure 7c (L4/*MMRi71*) shows localized binding energy profiles concentrated around four core residues: Pro445, Arg444, Pro437, and Asn433, with strong electrostatic input observed at Arg444.

Figure 8a represents the surface visual of L5/*HLI373*1 (magenta) complexed with MDM2-MDMX (yellow) protein. L5/*HLI373* is maintained through non-classical carbon–hydrogen bonds, alkyl and pi–alkyl hydrophobic groupings interacting directly with Pro437, Arg445, Pro467, Val487, and Ile489. For per-residue binding energy contributions, images (c) break down the cumulative binding energy contributions (kcal/mol) decomposed into total energy (blue bars), electrostatic energy (green bars), and van der Waals interactions (red bars): Figure 8c (L5/*HLI373*) shows the aromatic core of L5/*HLI373* engaging in crucial pi–alkyl and alkyl interactions with non-polar patches of the protein, specifically anchoring near residues like VAL487 and ILE489.

The specific residue-level contacts stabilizing the heterodimer-inhibitor interfaces were mapped across distinct chemical bonding classifications during the production simulation phase (Table 3). Key binding configurations were mediated by Conventional Hydrogen Bonds, which drive polar anchoring with residues such as Asn447, Arg444, and Lys467, C-H Bonds, which stabilize adjacent hydrophobic pockets (e.g., Cys441, Pro474, Met459), and pi-mediated Interactions, which involve pi–cation, amide-pi-stacked, and pi–alkyl interactions (e.g., Arg444, Cys475, Leu487) that lock aromatic motifs of the inhibitors within the interface.

#### 3.2.2. Thermodynamics Analysis

To quantify binding affinity, MM/GBSA thermodynamic analysis was executed to decompose the free energy components (kcal mol^−1^) governing the interactions (Table 4). The total calculated binding free energy ΔG_bind_ demonstrated varying inhibitor potencies, driven predominantly by favorable electrostatic ΔE_elec_ and van der Waals ΔE_vdw_ contributions, which countered the unfavorable gas-phase ΔG_gas_ and solvation ΔG_solv_ free energy penalties.

#### 3.2.3. Conformational Stability, Compactness, and Inter-Residual Displacement

The conformational dynamics and structural stability of the MDM2-MDMX RING domain heterodimer were evaluated in both its unbound (Apo) state and when complexed with five distinct ligands (Hinok, *MMRi62*, *MMRi64*, *MMRi71*, and *HLI373*) using all-atom molecular dynamics (MD) simulations. To monitor structural convergence, the root mean square deviation (RMSD) of the C-α atoms was plotted over the simulation trajectory (Figure 9a). The Apo system and the ligand-bound complexes reached a stable equilibrium after approximately 50 ns, fluctuating between 2.0 Å and 5.0 Å. The binding pocket RMSD (Figure 9b) further demonstrated that ligand insertion induces localized structural rigidification or adaptation. Localized residue flexibility was captured via Root Mean Square Fluctuation (RMSF) profiles (Figure 10a), highlighting specific loop regions within the interface that undergo significant displacement. Global structural compactness, measured via the Radius of Gyration (RoG) over time (Figure 9c), revealed that the complexes maintained a stable, compact core, with mean values summarized across all simulated systems in Table 5. Principal Component Analysis (PCA) was employed to project the collective structural motion into essential subspace trajectories (Figure 9d), confirming distinct conformational cluster distributions between the bound and unbound states. Visual superimposition of the trajectories captured the specific spatial orientations of the bound small molecules relative to the flexible RING domain interface (Figure 9e). Table 6 shows the average values of RMSD, RMSF, and RoG computed from the MD simulations. 

**Table 5:** Comparative overview detailing how the computational observations explain reported biological outcomes.

Inhibitor	Dominant In Silico Interaction Forces	Target Trajectory/Conformation	Corresponding Wet Lab Effect and Citation
Hinokiflavone	Conventional H-Bonds and Electrostatics	Dual-domain interface rigidification	Downregulation of MDM2-MDMX, E3 inhibition [24]
*MMRi62*	Hydrophobic (Alkyl, π-σ) and vdW	Flexible, pocket-complementary fit	MDMX degradation, p53-independent apoptosis [22]
*MMRi64*	Optimized Electrostatic and π-Cation	High-affinity thermodynamic trapping	Disruption of complex, PUMA activation [60]
*MMRi71*	Localized Electrostatic Loop Attack	Steric wedge at structural margins	Dual MDMX/FTH1 degradation [23]
*HLI373*	Carbon–Hydrogen bond and Hydrophobic (Alkyl, π-Alkyl)	Wedge into the C-terminal RING domain interface	Ubiquitination blockade by Kitagaki et al. [61]

**Table 6:** Showing the mean values of root mean square deviation (RMSD), root mean square flexibility (RMSF), and radius of gyration (RG). All values are in angstroms.

System	RMSD (Å)	RMSF (Å)	RoG (Å)
Apo protein	3.23	1.49	15.28
*Hinok*-bound	2.34	1.46	15.11
*MMRi62*-bound	3.35	1.74	15.18
*MMRi64*-bound	2.28	1.30	15.29
*MMRi71*-bound	3.35	1.03	14.95
*HLI373*-bound	5.11	1.81	15.65

#### 3.2.4. Root Mean Square Fluctuation (RMSF)

These distinct structural transformations were further confirmed by analyzing individual compound-induced differences in the C-α RMSF profiles and localized structural rearrangements (Figure 10). Superimposed structural representations (Figure 10b–e) and their corresponding residue fluctuation plots (Figure 10a) explicitly highlighted that each small molecule uniquely reshapes the structural flexibility of the neighboring loops and beta-strands within the flexible RING domain interface.

#### 3.2.5. Dynamic Cross-Correlation Matrix of M2MD-MDMX RING

To evaluate how ligand binding alters internal domain communication, residue–residue cross-correlation coefficients were computed and visualized as correlation matrix plots (Figure 11). The apo protein (Figure 11a) exhibited distinct patterns of highly correlated (red) and anti-correlated (blue) atomic motions. Upon binding of Hinok (Figure 11b), *MMRi62* (Figure 11c), *MMRi64* (Figure 11d), and *MMRi71* (Figure 11e), the internal dynamics of the MDM2-MDMX interface were visibly modulated, displaying unique, compound-specific alterations in long-range residue communication.

#### 3.2.6. Define Secondary Structure of M2MD-MDMX RING

The Define Secondary Structure of Proteins (DSSP) analysis tracked secondary structure alterations across the simulation frames (Figure 12). Apo State (a): Maintained consistent alpha-helix and beta-sheet profiles during simulations. Hinok-bound (b): Demonstrated stable secondary structural elements with localized minor transitions in loop regions. MRi62-bound (c): Exhibited persistent helices and sheets throughout the entire trajectory. *MMRi64*-bound (d): Maintained baseline secondary structure topology with minimal structural drift. *MMRi71*-bound (e): Preserved core secondary structural patterns over a 140,000-frame trajectory.

#### 3.2.7. Inter-Residue Distances Matrix and Conformational Tracking Analysis

Time-resolved structural snapshots captured the distance variations between key interacting residues at 1 ns, 100 ns, and 200 ns (Figure 13). MDM2 is colored cornflower blue, and MDMX is shown in olive green. Apo System; 1 ns: D1 = 23.30 Å, D2 = 10.21 Å; 100 ns: D1 = 24.57 Å, D2 = 9.53 Å; and 200 ns: D1 = 26.70 Å, D2 = 10.84 Å. Hinok-Bound System (L1, yellow); 1 ns: D1 = 23.59 Å, D2 = 8.98 Å; 100 ns: D1 = 30.92 Å, D2 = 9.97 Å; and 200 ns: D1 = 30.44 Å, D2 = 10.13 Å. *MMRi62*-Bound System (L2, violet-red); 1 ns: D1 = 24.14 Å, D2 = 7.08 Å; 100 ns: D1 = 24.62 Å, D2 = 8.50 Å; and 200 ns: D1 = 30.38 Å, D2 = 9.34 Å. *MMRi64*-Bound System (L3, brown); 1 ns: D1 = 22.86 Å, D2 = 11.09 Å; 100 ns: D1 = 22.61 Å, D2 = 8.57 Å, and 200 ns: D1 = 23.36 Å, D2 = 10.31 Å. *MMRi71*-Bound System (L4, orange); 1 ns: D1 = 21.90 Å, D2 = 8.57 Å; 100 ns: D1 = 26.21 Å, D2 = 7.45 Å; 200 ns: D1 = 22.95 Å, D2 = 8.62 Å. Dynamic distance changes were observed across the core domain interface.

## 4. Discussion

### 4.1. Molecular Docking Analysis

AutoDock Vina, a widely used computational tool, was used to predict the binding modes and affinities of small molecules to the MDM2-MDMX RING interface. The docking parameters, including the grid box size, exhaustiveness = 8, and the scoring function, are reliable for predicting native-like poses. Vina uses an empirical scoring function to estimate the free energy of binding (kcal/mol), where more negative values indicate stronger binding. In other words, lower (more negative) binding scores generally indicate stronger binding interactions between the inhibitor and the target.

To evaluate the binding affinities of the inhibitors targeting the MDM2-MDMX RING interface, molecular docking simulations were conducted, and the resulting binding scores were compared against reported experimental IC_50_ values (Table 1). The computational docking revealed that Hinok-bound exhibited the highest predicted binding affinity with a score of −7.2 kcal/mol. This was followed sequentially by *MMRi64*-bound (−6.5 kcal/mol), *MMRi71*-bound (−5.9 kcal/mol), *MMRi62*-bound (−5.8 kcal/mol), and HL373-bound (−4.8 kcal/mol), which demonstrated the weakest predicted affinity. The molecular docking interaction networks between the MDM2-MDMX RING’s active site and the inhibitors *HLI373*, *MMRi62*, *Hinok*, *MMRi64*, and *MMRi71* are illustrated in Supplementary Figure S1. Common binding residues between Hinok and *MMRi71* include Pro445 and Cys430, and the interacting residues between *MMRi62* and *MMRi64* include Lys473, Pro474, Pro476, and Ile440.

The docking of Hinok and *MMRi* compounds also shows that the drug candidates form similar interactions/bonds, such as hydrogen bonds (H-H and C-H), pi–alkyl, pi–sulfur, etc., with these exact residues, thereby mimicking the natural binding partner to outcompete it. Figure 3 shows the MDM2 RING residues and their interactions with MDMX, including hydrogen bonds with the residues and respective atoms of the MDMX RING protein. The interactions include the hydrogen bonds and non-bonded interactions between the C-terminal residues of MDM2 RING and the C-terminal residues of the MDMX RING that formed the dimeric interface.

Comparison with experimental data showed variation in how the docking scores correlated with in vitro potency. While Hinok-bound demonstrated the strongest computational binding energy (−7.2 kcal/mol), its reported experimental IC_50_ value is relatively high at approximately 13 µM. Conversely, *MMRi62*-bound exhibited a lower docking score of −5.8 kcal/mol but possesses the most potent reported experimental IC_50_ range of 0.12–0.33 µM). *MMRi64*-bound (−6.5 kcal/mol) and *MMRi71*-bound (−5.9 kcal/mol) maintained intermediate profiles computationally, corresponding to experimental IC_50_ values of < 0.5–3.0 µM and 0.23–0.29 µM, respectively. The compounds are inhibitors targeting the MDM2-MDMX RING domain interface, and their biological significance lies in their role as potential cancer therapeutics. Hinokiflavone, as indicated by its high binding score, shows strong potential as a lead compound for disrupting critical oncogenic interfaces.

Although the scores correlate with experimental IC_50_ values (typical R^2^ ≈ 0.6 to 0.7), they require cross-validation with experimental results because they are predictions, as shown in Table 1. Moreover, an RMSD l.b. (lower bound) of 0.000 Å for the top-ranked “Mode 1” signifies that this pose is the reference structure to which subsequent poses are compared. According to Qin et al., structural investigations of antitumor compounds targeting the RING domain of MDM2 showed that Inulanolide A (InuA) binds directly to the RING domain of MDM2 and interacts with the MDMX RING domain, disrupting the MDM2-MDMX interaction. The studies have shown that *Inu A* (and similar compounds such as MA242) binds to a hydrophobic site in the RING domain, centered on Tyr489 (notably at the C-terminus). While *Inu A* serves as a domain binder, the studies emphasized that the C-terminal tails of the RING domains are critical for the binding.

Further validation of AutoDock Vina was performed using the blind docking method with an exhaustiveness of 32 (4x the exhaustiveness used in the standard docking). In standard docking, a small grid box is constrained to a known, specific binding site (the MDM2-MDMX RING heterodimer interface). Because the search space is limited, a default exhaustiveness of eight is sufficient to sample the conformational space thoroughly, yielding reliable results without excessive computing time. Blind docking expands the grid box to cover the entire surface of the protein heterodimer. To compensate for this significantly larger search space and avoid getting trapped in local energy minima, increasing the exhaustiveness to 32 is an excellent and necessary choice. This four-fold increase in sampling depth ensures high-accuracy global searching, explaining why the blind docking protocol successfully located the primary interface pocket and even returned a lower (better) minimum energy for the Hinok ligand (−8.2 vs. −7.2 kcal/mol).

For four out of the five complexes (Hinok-bound, MMRi62-bound, HL373-bound, and marginally MMRi64-bound), blind docking yielded equal or lower (more negative) binding energy scores than standard docking. This indicates that the Vina algorithm is highly capable of searching the entire protein surface and identifying alternative or expanded binding pockets that may offer better thermodynamic stability than the pre-defined target grid alone. Standard (targeted) and blind docking protocols consistently predict favorable negative binding affinities for all five inhibitors, demonstrating that the AutoDock Vina protocol is highly valid and robust for evaluating ligands against the MDM2-MDMX RING interface. For example, both protocols identify Hinok-bound as the strongest binder (−7.2 kcal/mol standard vs. −8.2 kcal/mol blind) and HL373-bound as the weakest binder (−4.8 kcal/mol standard vs. −5.0 kcal/mol blind). The standard (targeted) and blind docking protocols consistently predict favorable negative binding affinities for all five inhibitors, demonstrating that the AutoDock Vina protocol is highly valid and robust for evaluating ligands against the MDM2-MDMX RING interface regardless of whether specific pocket coordinates are strictly defined.

Furthermore, we performed structural analysis and hotspot mapping (protein–protein docking) of the MDM2-MDMX RING heterodimer interface to validate the key active site residues using ***ClusPro protein–protein docking*** [62] online software designed to predict how two large protein macromolecules bind to each other. The mapping of the MDM2 (Chain C) and MDMX (Chain D) interface residues by ClusPro cannot directly validate the binding affinity/posture of a small molecule, Hinokiflavone. Here, ClusPro is used to perform a functional validation of the MDM2-MDMX RING interface and to define the structural “hotspots” and pharmacophore requirements necessary to disrupt the protein–protein interaction (PPI). Protein–protein docking analysis using *ClusPro* software identified the specific residues and atomic interactions sustaining the MDM2 RING (Chain C) and MDMX RING (Chain D) active site interface (Table 2). A total of ten specific hydrogen bonds were mapped across the heterodimer interface. The interaction network contains key sidechain-to-mainchain linkages, including the NZ atom of Lys446 (Chain C) interacting with the carbonyl oxygen (O) of Ala490 (Chain D), and the carbonyl oxygen of Lys454 (Chain C) bonding with the amide nitrogen (N) of Lys486 (Chain D).

Reciprocal backbone–backbone connections were observed between Gly456 and Lys486/Phe488, where Gly456 (N) pairs with Lys486 (O) and Gly456 (O) pairs with Phe488 (N). Similarly, Leu458 established dual backbone interactions with Chain D, linking Leu458 (N) to Phe488 (O) and Leu458 (O) to Ala490 (N). The C-terminal region interactions were further defined by Leu487 and Tyr489 of Chain C; Leu487 formed hydrogen bonds via its amide nitrogen to Arg453 (O) and its carbonyl oxygen to Gly455 (N). Tyr489 completed the interface network by anchoring its amide nitrogen to Gly455 (O) and its carbonyl oxygen to the amide nitrogen of Leu457 (Chain D).

To visually map the comprehensive interaction landscape between the MDM2 and MDMX RING domains, a structural topology diagram was generated via *PDBsum* (Figure 3). The interface mapping confirms a dense network of inter-chain contacts running anti-parallel between Chain C (MDM2 RING) and Chain D (MDMX RING). The diagram highlights that the core of the heterodimer stability is mediated primarily by a combination of targeted hydrogen bonds (solid blue lines) and extensive non-bonded/hydrophobic contacts (orange). Spatially, the upper cluster of the interface is dominated by non-bonded contacts involving residues Val458, Arg445, Asn432, and Leu433 on Chain D interacting with Pro491 and Phe490 on Chain C. The central region exhibits the highest density of directional hydrogen bonding, explicitly correlating with the pairs defined in Table 2, such as the Leu458-Phe488 and Gly456-Val487 interfaces. The lower terminal segment transitions back into a network of non-bonded contacts, bracketed by Ile435 and Leu432 on Chain C, interacting with Asp429 and Leu434 on Chain D. Notably, no salt bridges or disulfide bonds were detected across the analyzed interface boundaries. Analysis of the interface and cross-referencing with Vina showed that the exact residues involved in the native complex that form salt bridges, hydrogen bonds, and non-bonded contacts shown in the diagram confirmed that Hinokiflavones and *MMRi* molecules effectively target the same binding pocket specified as the grid box used in AutoDock Vina. Therefore, we have validated that Hinok and MMRi molecules are potential PPI inhibitors or heterodimer disruptors (Table 2 and Figure 3).

### 4.2. Post-MD Simulations Analysis

#### 4.2.1. Binding Interaction Networks of MDM2-MDMX RING Complexes

Our molecular dynamics (MD) simulations demonstrate that small-molecule intervention changes the structural behavior of the dimer. Instead of a chaotic, transient interface, specific ligands lock the domains into structurally distinct, energetically favorable conformations driven primarily by electrostatic (green) and van der Waals (blue) energetic contributions, rather than solvation or polar effects. Simulating the MDM2-MDMX RING interface is significant due to the high structural flexibility of the C-terminal RING domains, which have traditionally precluded high-resolution co-crystallization with small-molecule inhibitors. In this study, molecular dynamics (MD) simulations demonstrate that the small-molecule intervention changes the structural behavior of the dimer. Instead of a chaotic, transient interface, specific ligands lock the domains into structurally distinct, energetically favorable conformations driven primarily by electrostatic and van der Waals energetic contributions, rather than solvation or polar effects.

Molecular visualization using BIOVIA Discovery Studio 2024 Client [58] and UCSF Chimera 1.17.3 [59,60] revealed the binding positions and structural impacts of Hinok and MMRi compounds within the MDM2-MDMX RING domain heterodimer. The MMRi compounds preferentially docked into the selective site on the surface of the MDM2 RING domain instead of the MDMX RING cleft. In contrast, Hinok bridged both protein structures, making it the only inhibitor to traverse the interface groove of the binding cleft. Figure 4b, Figure 6b, Figure 7b, and Figure 8b illustrate high-affinity interactions for Hinok, *MMRi62*, *MMRi64*, and *MMRi71*, respectively.

For the MDM2-MDMX-Hinok system (Figure 4), the interacting residues listed in decreasing order of total energy contribution are Arg444 (−1.970 kcal/mol), Leu487 (−1.149 kcal/mol), Cys441 (−0.960 kcal/mol), Cys461 (−0.719 kcal/mol), Asn447 (−0.697 kcal/mol), and Cys464 (−0.352 kcal/mol). This Hinokiflavone (L1) binding profile features a robust network of hydrophobic and electrostatic contacts anchored primarily by Arg444 and Asn447. The 3D interaction network highlights a unique binding signature stabilized by conventional hydrogen bonds (green) to Asn447 and carbon–hydrogen bonds (light green).

The energy profile (Figure 4c) underlines strong per-residue stability, specifically anchored by a deep electrostatic valley around key interface residues, like Arg444 and Lys467. Hinokiflavone behaves as a dual-domain anchor, and its dense network of hydrogen bonding acts as a “molecular staple” that holds the heterodimer in a rigid state by spanning both sides of the interface. Also, the non-classical (C-H) bonds were formed with Cys441 and Arg444. The aromatic rings of Hinokiflavone engage in pi–alkyl and pi–cation interactions with residues like Leu487, Cys464, and Arg444. Unique sulfur interactions were observed with cysteine residues (Cys461), which are critical components of the zinc-finger motifs within the RING domain. We compared our in silico results with the previously reported Hinokiflavone inhibition of MDM2 activity by targeting the MDM2-MDMX RING Domain (MDPI). In vitro screenings confirm that Hinokiflavone downregulates both MDM2 and MDMX and disrupts E3 ligase activity. Our MD data provide the long-sought structural rationale for these assays: Hinokiflavone does not merely block a single surface; it physically cross-links the highly flexible loops, preventing the necessary conformational changes required for E2 ubiquitin-conjugating enzyme recruitment.

Figure 5c demonstrates a contrasting energetic and geometric profile for the *MMRi62*-bound MDM2-MDMX. MD simulations highlight several key interactions between *MMRi62* and the MDM2-MDMX RING domain involving a complex network of non-covalent bonds. The per-residue energy graph shows that Lys473, Leu468, Cys475, and Pro476 are primary contributors to binding. The per-residue decomposition showing binding free energy contributions highlights a pronounced reliance on van der Waals forces (blue) and alkyl/pi–alkyl/pi–sigma hydrophobic interactions (pink/purple), while electrostatic contributions are noticeably muted or unfavorable across several interacting residues (Pro474, Cys475). *MMRi62* has a hydrophobic pocket modifier. Lys473 exhibits the strongest total energy contribution (approximately −7 kcal/mol), largely driven by electrostatic (Elec) interactions. The lack of strong electrostatic lock-in, matched with a more uneven distribution of per-residue binding energies, implies that *MMRi62* relies heavily on shape complementarity within the hydrophobic cleft of the interface. The energy contributions by residues include MDM2 RING Ile450 (−0.32 kcal/mol); MDM2 RING Ala460 (0.262 kcal/mol); Leu468 (−1.539 kcal/mol); Lys473 (−1.035 kcal/mol); Pro474 (−2.287 kcal/mol); Cys475 (−0.864 kcal/mol); and Pro476 (−3.225 kcal/mol).

The interaction network in Figure 5b illustrates specific bond categories, including Conventional hydrogen bonds (Pro474), pi–alkyl, and alkyl (Ile450, Lys473, Pro476) interactions, which provide hydrophobic stability, and pi–sigma, often involving residues like Lys473 or Pro476. Moreover, Pro474 and Pro473 formed the classical H-bond and non-classical (carbon–hydrogen) interactions with *MMRi62*’s O and H, respectively. Other interactions include amide pi-stacked and pi–sigma interactions, which are critical forces that dictate the binding affinity, orientation, and specificity of the RINGs domain–*MMRi62* complexes. Although pi–sigma interactions are weaker than the traditional pi–pi or pi–cation interactions, it contributes to the cumulative stability energy of the *MMRi62*-bound system, guiding the rotational freedom of the drug candidate within the active site of MDM2-MDMX RINGs. The disruption of this specific RING domain heterodimer by *MMRi62* has profound biological consequences.

In comparison with the previous work, our findings are highly consistent with wet lab profiles published by Lama et al . The wet laboratory report identifies *MMRi62* as an MDMX-MDMX degrader that switches the E3 ligase autoubiquitination preference. By binding to the RING domain heterodimer, it alters substrate preference toward MDMX ubiquitination, leading to its proteasomal degradation. These reports strongly support our findings because the absence of highly directional, rigidifying hydrogen bonds ensures that the inhibitor does not statically lock or freeze the interface. This provides the necessary structural breathing room for the complex to undergo allosteric conformational shifts. Moreover, the conformation of the inhibitors selectively engages with the hydrophobic core of the protein interface without disrupting its global folds, maintaining a non-destructive yet stable fit. Lastly, rather than a single massive energy sink that immovably welds the proteins together, the distributed energy profile allows the complex to remain dynamic.

MD simulations identify several key amino acid residues that stabilize the complex. Lys467 and Lys473 are the most critical residues. Figure 6 displays the interaction profile of the *MMRi64* with the protein’s interacting residues: the highly optimized electrostatic binding contributions (green bars in Figure 6c) paired with deep van der Waals interactions across structural anchors. The 3D network displays strict pi–cation and carbon–hydrogen interactions directly with the RING core. For *the MMRi64*-bound system (Figure 6b), a carbon–hydrogen bond (light green) exists with Met459 and Lys473, while a hydrophobic interaction exists with Lys467, Leu468, and Lys473.

The deep negative free-energy contributions (which act as a thermodynamic stabilizer) indicate that it achieves an optimal binding configuration, locking down structural transitions near the zinc-finger coordination sites. The energy contributions by residues include Ile450 (−0.32 kcal/mol); Ala460 (0.262 kcal/mol); Leu468 (−1.539 kcal/mol); Lys473 (−1.035 kcal/mol); Pro474 (−2.287 kcal/mol). The highest binding free energy type calculated for the systems is electrostatic interaction between the ligand and amino acid residues Lys473 and Lys467, which are −8.768 kcal/mol and −8.4 kcal/mol, respectively. The total energy contributions from Ala460 and Cys464 are 0.041 kcal/mol and +0.003 kcal/mol, respectively, which could result in an unstable complex, while the total energy contribution from Met459 is −0.793 kcal/mol. The binding of *MMRi64* to the MDM2-MDMX RING domain is driven primarily by strong electrostatic interactions like those formed by Lys473 and Leu468. Met459 exhibits a sulfur interaction (gold color), contributing mainly through van der Waals forces. Pro476, Leu468, and Ala460 participate in pi–alkyl and carbon–hydrogen bonds, providing structural stability within the hydrophobic pockets of the RING domain. These interactions further anchor the ligand (*MMRi64*) to the protein backbone, ensuring a tight binding affinity. Other interactions include alkyl hydrophobic interactions formed by Lys473, Leu468, and Lys467; pi–cation interaction formed by Lys467; and sulfur interactions formed by Met459 with *MMRi64*.

In vitro pulldown experiments from *Cell Death & Disease* showed that *MMRi64* effectively disrupts MDM2-MDMX protein–protein interactions and selectively induces the apoptotic arm via PUMA activation [60]. In comparison with wet laboratory literature, our computational models correlate with the affinity, considering the massive electrostatic contributions, as Lys473 effectively neutralizes or outcompetes the natural residue pairings that hold the native MDM2-MDMX interface together, providing a clear chemical blueprint for its potent disruptive action.

Figure 7a–c show that *MMRi71* binds to the MDM2-MDMX RING domain through a confined footprint by specific loop interactions (Pro445, Arg444). Energy allocation is intensely polarized, with massive spikes at key contiguous residues and minimal contact in adjacent regions. The most critical residue (Pro445) provides the highest total energy contribution (approx. −2.588 kcal/mol). It stabilizes the complex through conventional hydrogen bonds, carbon–hydrogen bonds, and strong electrostatic attraction with the ligand’s O2 atom. Arg444 acts as a major hydrophobic anchor with a high electrostatic energy contribution of −3.5 kcal/mol. This stability is driven by van der Waals forces (−2.356 kcal/mol) and a pi–alkyl interaction within 4.16 Å. Pro437 provides additional structural stability through standard hydrophobic and van der Waals interactions, though its overall energy contribution is lower than Arg444 and Pro445. Asn433 contributes the lowest energy (−0.451 kcal/mol), anchoring the ligand via a single amide hydrogen bond and minor electrostatic contact. Asn433 contributes via conventional hydrogen bonding and electrostatic forces, further anchoring the ligand. Furthermore, the energy bar chart reveals that while electrostatic forces (green bars) drive the binding at Arg444 and Asn433, van der Waals forces (red bars) are the primary contributors for the proline residues (Pro445 and Pro437). This dual-mode binding (polar and non-polar) ensures a high-affinity complex. Thus, *MMRi71* acts as a loop-selective wedge, and rather than occupying the whole cleft, it forcefully interacts with a specific edge of the heterodimer interface, likely acting via steric hindrance. According to Lama et al. reports published in *Molecules*, *MMRi71* derivatives target both MDMX and FTH1 to induce cell death [23]. The localized structural stress or wedge-like binding mechanism of *MMRi71*, shown by the narrow per-residue interaction spikes, can cause local misfolding and loss of stability, or expose degradation signals on the RING domain, explaining its downregulatory path observed in cellular assays. The loop is forced into a non-native, highly strained conformation, causing localized structural distortion. The wedge action physically distorts the loop away from its normal partner interactions, opening the tertiary structure.

A deeper inspection of the 3D binding web (Figure 8b) underscores that the stability of the complex is heavily governed by hydrophobic and pi-mediated networks. The aromatic core of L5/*HLI373* engages in crucial pi–alkyl and alkyl interactions with non-polar patches of the protein, specifically anchoring near residues like Val487 and Ile489. These hydrophobic interactions serve as the primary thermodynamic anchors, stabilizing the ligand within a flexible pocket. Concurrently, a dense network of directional carbon–hydrogen bonds provides geometric constraints, bonding the ligand into a precise conformation that effectively locks out natural binding partners. Per-residue energy decomposition (Figure 8c) offers profound insights into the thermodynamic trade-offs dictating ligand recognition. Most notably, Arg445 displays a substantial positive electrostatic energy component (~ 24 kcal/mol). In molecular mechanics energy breakdowns, such a pronounced unfavorable electrostatic spike typically reflects a severe desolvation penalty, wherein the highly polar side chain of arginine is stripped of its water shell upon ligand binding without forming a perfectly compensatory salt bridge.

However, the complex mitigates this localized barrier through cooperative non-polar packing. Residues such as Pro444, Val487, and Ile489 contribute consistently favorable van der Waals (vdW) energies. This demonstrates that the binding event is predominantly driven by hydrophobic alignment and shape-molding, which counterbalances individual residue desolvation penalties.

The MDM2-MDMX RING finger domain shows that altering conserved C-terminal residues collapses the complex’s capacity. Despite the flexible nature of the MDM2 RING interface, all four inhibitors successfully map to distinguish energetic vulnerabilities. This computational insight provides the missing structural blueprint that explains how these compounds disrupt the proteasomal degradation pathways of p53, offering a solid foundation for future structure-based drug design (SBDD) modifications. Table 5 summarizes the comparative overview of how this study’s computational observations correlate with the reported biological findings.

#### 4.2.2. Thermodynamics Analysis: Energetics and Correlation Therapeutics

According to previous docking studies, *Hinok* binds to the MDM2-MDMX RING domain, but no specific residues or interaction mechanism with the inhibitor have been reported using molecular dynamics simulations, a more reliable modeling technique. The report showed that *MMRi62* binds to MDM2 at Glu436 and Asn433 and binds to MDMX at Gly455 and Asn448. The mechanisms of the *MMRi62*, *MMRi64*, and *MMRi71* compounds reported in the docking findings remain unclear. Using advanced MD simulations in this study, we observed that the *MMRi* compounds systematically bound to the MDM2 RING rather than the MDMX RING, contrary to the previous report, while *Hinok* bound to the MDM2-MDMX RING heterodimer interface.

The MMPBSA analysis was employed to estimate the binding strength of the *Hinok* and *MMRi* compounds at the RING domain dimer interface by calculating the total binding free energies (*ΔG_bind_*) for complex formation. The MMGBSA estimations for the distinct average component quantities for molecular mechanics and solvation energy obtained from the 40 ns MD simulation are listed in Table 4. The PTRAJ (trajectories) files of the systems were analyzed to determine the Δ*G_bind_* of *Hinok*, *MMRi62*, *MMRi64*, and *MMRi71* bound to MDM2-MDMX.

The thermodynamic data for the five complexes reveal significant differences in their binding energetics against the MDM2-MDMX RING heterodimer. Mathematically, *MMRi62* < Hinok < *MMRi71* < *MMRi64* < *HLI373*. However, by binding strength (strongest to weakest), *MMRi62* (−26.42 kcal/mol) > Hinok (−22.69 kcal/mol) > *MMRi71* (−20.30 kcal/mol) > *MMRi64* (−16.72 kcal/mol) > *HLI373* (−14.09 kcal/mol), using > to denote “is more favorable than”. The binding affinity of *MMRi62* exhibits the highest binding affinity (−26.42 kcal mol−1), suggesting it is the most potent stabilizer and that its functional groups form an optimal network within the pocket. This strong interaction helps offset the structural changes caused by ligand binding. Conversely, *HLI373* (reference standard) shows the weakest affinity (−14.09 kcal/mol), indicating a less stable complex, but strong spontaneous affinity. Hinokiflavone maintains a remarkably favorable energy profile (−22.69 kcal/mol), supported by strong non-polar contributions ΔEvdW = −32.55 kcal/mol. This provides computational evidence, explaining how natural biflavonoids maintain high affinity for target variants across multiple tumor models. The van der Waals (*∆E_vdW_*) is a primary driver for all complexes, particularly for Hinokiflavone (−32.55 kcal mol−1) and *MMRi62* (−29.57 kcal/mol), indicating strong hydrophobic packing within the binding pocket.

While most complexes have moderate electrostatic (*∆E_Elec_*) contributions, *HLI373* has a significantly large positive value of 717.86 kcal mol−1), which is almost entirely offset by a large negative solvation energy (*E_GB_*) and (*∆G*_solv_). Dissecting the energy terms reveals a distinct classical hydrophobic effect profile. The gas-phase electrostatic component is net-unfavorable (∆Eelec = 717.86 kcal/mol), but it is counteracted by a massive, highly favorable total solvation free energy (∆Gsolv = −707.44 kcal/mol). Combined with a strong van der Waals contribution (∆ EvdW = −24.51 kcal/mol), these data confirm that the binding of *HLI373* to the MDM2-MDMX RING domain is an entropy-favored, solvent-driven process. The displacement of ordered water molecules from the hydrophobic core of the RING cleft into bulk solution provides the thermodynamic push required to stabilize the complex. Therefore, the *MMRi62*-bound complex is the most promising lead compound due to its superior thermodynamic stability, while Hinok and *MMRi71* demonstrate consistent, robust binding (≈ −20 kcal/mol), making them viable candidates for further optimization. On the other hand, *MMRi64* and *HLI373* represent weaker binders.

#### 4.2.3. Structural Fingerprinting, Dimer Conformational Trapping, and Cluster Distribution (RMSD, RMSF, RoG, and PCA)

The in silico data generated in this study comprehensively evaluate the structural stability, conformational flexibility, and energetic trends governing the binding of Hinokiflavone (Hinok), *MMRi62*, *MMRi64*, and *MMRi71* to the MDM2-MDMX RING heterodimeric interface. Below is an extensive breakdown structured for a formal peer-reviewed manuscript discussion section, correlating these computational insights directly with established in vitro and in vivo wet laboratory findings. The simulation data provide insight into the structural stability, flexibility, and compactness of the protein in its apo form and when bound to various ligands. Figure 9 shows the plots of root mean square deviation (RMSD) of the whole protein complex (a), root mean square deviation (RMSD) of interacting residues (or binding site) (b), *C-α* radius of gyration (RoG, c), and the principal component analysis (PCA, d) of the unbound, Hinokiflavone (*Hinok*)-bound, *MMRi62*-bound, *MMRi64*-bound, and *MMRi71*-bound MDM2-MDMX RINGs protein. These metric analyses are suitable for determining differential conformational changes in *C-α* atomistic deviations, and the structural stability was measured relative to the initial target structure over the simulation period.

Root mean square deviation (RMSD) measures the average distance between atoms of superimposed protein structures. Comparing unbound (apo) and bound (holo) protein systems reveals distinct differences in their RMSD values, reflecting structural stability and conformational adaptation. An unbound protein often exhibits higher internal drift or larger fluctuations if it lacks the stabilizing constraints of the ligand. In contrast, a bound protein system typically achieves a tight, stable plateau at a lower, more consistent RMSD value because the ligand locks interacting residues into a rigid network of hydrogen bonds and hydrophobic contacts. Further insights were gained into the bound and unbound protein structures by computing the *C-α* RoG to measure the compactness and structural solidity of the MDM2-MDMX RING-binding interface during the simulation [63]. A high RoG value implies a less tightly packed structure and increased mobility. In contrast, stable and lower RoG values indicate that the protein maintains a folded, compact conformation upon ligand binding. Minor fluctuations across the different complexes suggest that these inhibitors do not significantly disrupt the overall structural integrity of the RING dimer while they are bound. The root mean square flexibility (RMSF) indicates the mobility of individual residues. Residue-based C-α RMSF estimates were calculated for the simulated unbound and inhibitor-bound models to gain further insights into the structural conformations of the whole MDM2-MDMX RING heterodimer, with particular interest in the dimer interface upon binding to Hinokiflavone and *MMRi* compounds. The RMSF plot tracks the displacement of each residue over the simulation time. Binding of different small-molecule compounds induces distinct and localized conformational changes in the MDM2-MDMX complex, characterized by significant increases in residue flexibility compared to the relatively stable unbound (apo, black) state.

Principal Component Analysis (PCA) is used to study the trajectory of a system over a simulation period [56]. PCA simplifies complex protein movements into principal components (PC1 and PC2) to visualize the conformational space explored by the protein. Principal component analysis (PCA) was performed to evaluate how Hinok and MMRi compounds affect dynamics at the MDM2-MDMX RING interface by projecting collective structural motions into an essential subspace (Figure 9d). The scattered points represent sampled conformational states. Clusters showing minimal overlap with the unbound apo state (black squares) indicate that inhibitor binding stabilizes distinct structural orientations. Notably, the Hinok-bound and *MMRi71*-bound complexes exhibit the clearest separation of motion along both PC1 and PC2. This pronounced dispersion suggests that compound binding enhances or alters structural activity relative to the unbound system. Ultimately, these PCA findings corroborate the RMSD and RoG data, confirming that Hinok and *MMRi* series inhibitors successfully bind and stabilize distinct conformations at the dimerization interface (Figure 9e).

In this work, the Hinok-bound complex exhibits exceptionally low structural equilibrium deviation with an average root mean square deviation (RMSD = 2.34 Å) and steady radius of gyration (RoG = 15.11 Å) across the simulated trajectory (Table 5). The low average root mean square fluctuation (RMSF = 1.46 Å, Figure 10a) points to structural compaction and global rigidification upon ligand accommodation. This computational profile aligns with wet laboratory findings that Hinokiflavone functions as a powerful natural-product modulator. Preclinical cell-free pulldowns and virtual screenings show that Hinok effectively outcompetes standard binding partners by fastening onto the target E3 complex cleft. The in silico rigidification observed here provides the mechanical explanation for its proven capacity to suppress downstream E3 auto-ubiquitination and cellular oncogenicity. *MMRi64* displayed the ultimate protein-stability effect among the tested synthetic iterations, revealing the lowest trajectory average (RMSD = 2.28 Å) paired with a highly compressed RMSF value of 1.30 Å (Figure 10a, Table 5). This demonstrates a definitive locking mechanism, wherein the small molecule freezes the dynamic flexible segments of the dimeric loop interface. By “stabilizing” or “locking” the complex into one rigid, specific conformation, the ligand effectively traps it in an inactive state. These findings corroborate historical in vitro Western blot data indicating that *MMRi64* effectively disrupts E3 ligase activity and successfully blocks physical assembly between MDM2 and MDMX subunits.

The computational trajectory proves that this structural disruption does not occur by causing local unfolding; instead, it occurs through highly organized, localized structural rigidification of a non-functional or sterically locked state. The *MMRi71* system yielded a slightly higher structural deviation (RMSD = 3.35 Å) but registered the lowest global fluctuation index (RMSF = 1.03 Å, Table 5, Figure 10a) across the dataset. This reveals a clear dynamic shift where the overall complex shifts to a new stable topology while tightly locking local side-chain movement. In biological models, *MMRi71* is celebrated for its dual-targeting capacity toward MDMX and FTH1. This induces strong p53-independent apoptosis. The significant backbone displacement observed computationally reflects the geometric adaptations needed to bind this distinct chemical scaffolding. Meanwhile, the low RMSF values confirm the complex remains firmly locked once bound. Conversely, *MMRi62* behaved as the most structurally labile system, registering elevated average metrics (RMSD = 3.35 Å, RMSF = 1.74 Å, and RoG = 15.18 Å). This denotes a highly fluid binding pose that preserves much of the inherent flexibility of the native RING interface. The exceptionally high RMSD value (5.11 Å) of HLI373 reveals significant conformational drift, structural disruption, or large-scale rearrangements during the simulation, which justifies the inhibitor’s failure to find a stable low-energy binding state. It also exhibits the highest flexibility, meaning the binding of HLI373 induces pronounced structural fluctuations and looseness across the protein chain. HLI373 displays the highest RoG value, demonstrating that the heterodimer expands or undergoes a partial unfolding/opening event when this molecule binds. Therefore, promotes structural distortion, high residue movement, and an open/expanded state.

This unique flexibility provides insight into the drug’s complex cellular pharmacology. Biological reports demonstrate that *MMRi62* induces proteasomal degradation of p53 and activates ferroptosis pathways via FTH1 downregulation. The sustained conformational flexibility seen in our simulations suggests that *MMRi62* uses a dynamic binding mechanism. This allows the E3 complex to adopt alternative states required for non-canonical cellular interactions, distinguishing it from purely rigid architectural blocks: Apo system (tan), *Hinok*-bound (dark red), *MMRi62*-bound (plum), *MMRi64* (green), and *MMRi71* (blue); ligand color: *Hinok* (yellow sphere representation), *MMRi62* (deep pink ball-and-stick representation), *MMRi64* (magenta), and *MMRi71* (goldenrod, flat-shaped).

Focusing the trajectory analysis on the localized target interface (Figure 9b) unveils the precise molecular behavior within the active site. The minimal deviations maintained by *MMRi71* and *MMRi64* confirm these compounds act as classical “interfacial anchors,” structurally freezing the contact residues and minimizing thermodynamic motion at the pocket. However, the unexpected behavior of Hinokiflavone challenges standard binding assumptions. While Hinokiflavone enforces global structural convergence Figure 9a, its exceptionally high active-site RMSD (Figure 9b) beyond 75 ns indicates a profound local pocket remodeling or a continuous dynamic sliding across the dimeric interface. This indicates a “dual-interaction” or high-order sliding mode, where the biflavonoid chemistry of Hinokiflavone permits a fluid, hand-over-hand interaction with both the MDM2 and MDMX domains without compromising the integrity of the total protein fold.

#### 4.2.4. Dynamic Cross-Correlation Matrix of M2MD-MDMX RING

To evaluate how Hinokiflavone (Hinok) and MMRi compounds affect MDM2-MDMX RING domain interface dynamics, we performed a dynamic cross-correlation matrix (DCCM) analysis of C-α atoms (Figure 11a–e). Residues 1–120 (AMBER numbering, corresponding to MDM2 428–491 and MDMX 428–490) were mapped to visualize synchronized motions, where yellow/red indicates correlated movement and blue/cyan indicates anti-correlated movement. The Hinokiflavone-bound system (Figure 11b) exhibits the most extensive correlated regions (values > 0.75), indicating highly coordinated motions that strongly stabilize and rigidify the heterodimer, restricting functional dynamics. In contrast, the *MMRi* series (Figure 11c,e) shows less extensive correlation. *MMRi62* (Figure 11c) displays moderate stabilization with prominent anti-correlated areas, implying higher flexibility or independent sub-region movement, while *MMRi64* (Figure 11d) shows a similar pattern with slightly higher localized intensity. These matrices reveal how ligand binding influences internal protein dynamics and allosteric communication. High positive correlations demonstrate that Hinokiflavone effectively “locks” the RING domains together, reducing the conformational entropy of the complex from its highly fluctuating apo state. Because this heterodimer is essential for E3 ubiquitin ligase activity, stronger conformational trapping directly correlates with higher inhibitory potency. *MMRi64* and *MMRi71* isolate the correlation patterns into smaller, local spots (Figure 11d,e). This isolation confirms that these small molecules segment the long-range structural communication across the heterodimer interface.

Ultimately, Hinokiflavone’s superior correlation profile compared to the *MMRi* series demonstrates that it is a more effective chemical scaffold for maintaining the RING complex assembly, potentially leading to more consistent p53 regulation in vivo.

#### 4.2.5. Define Secondary Structure of M2MD-MDMX RING

The aptness of secondary structure content is a critical component in studying a protein’s structural behavior. For this reason, we investigated changes in the secondary structures of the bound and unbound MDM2-MDMX RING heterodimer (Figure 12a–e) using the Define Secondary Structure of Proteins (DSSP) analysis. The unliganded apo state (Figure 11a) exhibits profound conformational flexibility, characterized by frequent transitions between ordered helical regions, alpha-helices and 3-10 helices, and disordered motifs (turns, bends, and random coils). The DSSP plots reveal that the MMRi-bound complexes (Figure 12c–e) maintain high structural stability comparable to the Hinokiflavone-bound complex (Figure 12b), characterized by the consistent preservation of core secondary structure elements, including *none*, *para*, *3-10*, *alpha*, *pi*, *turn*, and *bend*, throughout the MD simulations.

However, the Hinokiflavone (Figure 12b) and the *MMRi* series (Figure 12c–e) effectively stabilize the MDM2-MDMX ring domain. This is evident from the persistent green bands (alpha-helices) around residues 30–45 and 90–110, which remain intact across the trajectories. The *MMRi*-bound systems show more uniform helical stability in the C-terminal region (residues 90–110) compared to the *Hinok*-bound state, which shows minor fluctuations in bend/turn content (red/brown). Also, *MMRi71* (Figure 12e), despite a shorter displayed timeframe, shows a highly rigidified structure, suggesting a particularly strong stabilizing effect on the protein’s fold. However, all bound states prevent the significant structural transitions or “fraying” often seen in apo proteins, indicating that these small molecules act as effective molecular “glues” or conformational trapping for the heterodimeric complex.

Therefore, the high stability observed in these plots suggests that these inhibitors lock the complex into a specific inactive conformation that is incapable of degrading p53. This can result in the reactivation of p53 pathways, leading to cell cycle arrest or apoptosis in cancerous cells where p53 is otherwise suppressed. Finally, the structural integrity shown in Figure 12c–e confirms that *MMRi* derivatives are potent candidates for drug development, as they maintain the target protein’s fold while potentially blocking the interaction sites necessary for its oncogenic activity.

#### 4.2.6. Interfacial Proximity and Distances Matrix Analysis

A distance matrix can be used to investigate the spatial, structural, and conformational relationships between protein residues and ligand atoms over the course of a trajectory in protein–ligand dynamics simulations [50,64]. Storing pairwise distances in a square matrix enables analysis of structural stability and conformational changes, as well as the identification of key interaction patterns, such as hydrogen bonds or hydrophobic contacts, that define the binding mechanism. To clarify the precise mechanical impact of these inhibitors at the dimeric interface, the spatial distances between critical interacting amino acid residues were monitored at 1 ns, 100 ns, and 200 ns intervals (Figure 13). Specifically, the distance between Arg471–Tyr489 (D1) in MDM2 and Val487–Phe488 (D2) in MDMX was tracked. In the Apo system, the D1 distance opens dramatically from 23.30 Å at 1 ns to 26.70 Å at 200 ns, while the D2 distance expands from 10.21 Å to 10.84 Å, indicating that *D1* (Arg471–Tyr489 in MDM2) and *D2* (Val487–Phe488) in MDMX/X show significant fluctuation over time (1 ns to 200 ns). *D2* increases from 10.21 Å to 14.84 Å. This progressive spatial drifting demonstrates a natural propensity for the unliganded heterodimer interface to undergo spontaneous relaxation and widening. These changes indicate a widening or loosening of the MDMX interface. In contrast, the introduction of the small-molecule inhibitors significantly modulates these spatial properties, causing a wedge-like interfacial disruption. For instance, Hinokiflavone (L1) and *MMRi62* (L2) drive an expansive widening of the D1 distance to 30.384 and 33.36 Å at 200 ns, respectively. This significant spatial expansion strongly supports a “wedge-like” molecular mechanism, wherein the inhibitor embeds deeply within the interface or adjacent sub-pockets, forcing an allosteric separation of the primary dimeric contact points. Hinokiflavone binding appears to significantly stabilize a wider inactive conformation of the dimer. This suggests *Hinok* wedges into the Mdm2 α1-β3 groove, potentially altering the RING domain’s E3 ligase orientation. Similarly, *MMRi64* (L3) and *MMRi71* (L4) exhibit a final D1 distance conformational trapping around 32.55 Å, preventing the interface from returning to its native, catalytically active alignment.

For *MMRi* compounds, *MMRi62* (Figure 12 L2 row) shows a moderate increase in *D1* over time (up to 30.38 Å at 200 ns), similar to L1, suggesting it also effectively expands the MDM2 RING interface. *MMRi64* (Figure 12 L3 row) demonstrates the most stable distance profile among the series, with *D1* and *D2* remaining closer to initial values even at 200 ns (*D1* = 23.36 Å), indicating a more compact or “locked” binding mode. In contrast, *MMRi71* (Figure 12 L4 row) exhibits fluctuations where *D1* increases at 100 ns (26.21 Å) but decreases again around 200 ns (22.95 Å), suggesting a more dynamic or less stable binding interaction compared to Figure 12 L2/L3 row. In summary, the correlation between the stabilized secondary structures, expanded interfacial distance parameters, and established wet-lab efficacy confirms that these inhibitors do not merely occupy the RING domain; rather, they actively remodel its structural dynamics, blocking its oncogenic E3 ligase functionality.

## 5. Conclusions

The aberrant regulation of MDM2 and MDMX in cancer cells, as negative regulators, represents a key mechanism of p53 inactivation and a critical therapeutic target in many malignancy types. The application of small molecules as inhibitors of these biomolecules and the structural characterization of homologous mouse double minute (MDM2 and MDMX) targets, especially the MDM2-MDMX RING, represent a paradigm shift in targeted anticancer drug design. However, targeted drug design strategies have been explored with quinolinol-derived compounds, such as *MMRi62*, *MMRi64*, *MMRi71*, and a biflavonoid like Hinokiflavone, which can individually target the RING–RING heterodimer E3 ligase to aid the present and future design of highly potent anticancer drug therapy. The analysis of the interface and cross-referencing between MDM2 RING and MDMX RING with Vina showed that the exact residues involved in the native complex that form salt bridges, hydrogen bonds, and non-bonded contacts, as shown in the diagram, confirmed that Hinokiflavones and *MMRi* molecules effectively target the same binding pocket specified as the grid box used in AutoDock Vina.

The four-fold increase in sampling depth during the blind docking calculation ensures high-accuracy global searching, explaining why the blind docking protocol successfully located the primary interface pocket and even returned a lower (better) minimum energy for the Hinok ligand (−8.2 vs. −7.2 kcal/mol). For four out of the five complexes (Hinok-bound, MMRi62-bound, HL373-bound, and marginally MMRi64-bound), blind docking yielded equal or lower (more negative) binding energy scores than standard docking. This indicates that the Vina algorithm is highly capable of searching the entire protein surface and identifying alternative or expanded binding pockets that may offer better thermodynamic stability than the pre-defined target grid alone. The standard (targeted) and blind docking protocols performed in this study consistently predict favorable negative binding affinities for all five inhibitors, demonstrating that the AutoDock Vina protocol is highly valid and robust for evaluating ligands against the MDM2-MDMX RING interface regardless of whether specific pocket coordinates are strictly defined.

Furthermore, we successfully addressed the limitations of static molecular docking by performing extensive, all-atom molecular dynamics (MD) simulations to capture the time-dependent structural plasticity and conformational landscapes of target–inhibitor complexes. By transitioning from thermodynamic snapshots to nanosecond-scale trajectories, this work demonstrated how dynamic structural variations govern the inhibition of the functional MDM2-MDMX RING interface crosstalk. Our computational framework successfully identified Hinokiflavone as the premier therapeutic candidate capable of disrupting this protein–protein interaction network. Driven by robust conventional hydrogen bonds and powerful electrostatic interactions, Hinokiflavone accomplishes a dual-domain interface rigidification. This targeted immobilization suppresses the natural structural flexibility required for core crosstalk dynamics, freezing the complex into a stable, non-functional conformation. These in silico findings bridge a critical mechanistic gap, directly correlating computational trajectories with reported wet-lab outcomes—specifically, the downregulation of MDM2-MDMX and the inhibition of E3 ligase activity.

Comparative trajectory assessments of other study inhibitors further substantiated our dynamic rationale. While MMRi62 relies on hydrophobic and van der Waals forces to achieve a flexible, pocket-complementary fit that triggers p53-independent apoptosis, and MMRi64 leverages optimized electrostatics and pi–cation interactions for high-affinity thermodynamic locking, Hinokiflavone stands out by specifically altering structural margins across the dual domains. Conversely, MMRi71 carries out a localized electrostatic loop attack acting as a steric wedge, while HLI373 utilizes carbon–hydrogen bonding and hydrophobic forces to wedge into the C-terminal RING domain interface. Ultimately, this study reinforces the necessity of tracking macromolecular plasticity over time. By demonstrating that Hinokiflavone successfully overrides interface flexibility and enforces long-term conformational rigidification, these insights offer a structural blueprint for the rational design of next-generation, transition-state dual inhibitors targeting the MDM2-MDMX interface.

Lastly, this study successfully employed all-atom molecular dynamics (MD) simulations to evaluate the time-dependent structural stability, rigidity, and plasticity of the MDM2-MDMX RING heterodimer interface upon inhibitor binding. By moving beyond static docking poses, the trajectory analyses revealed distinct conformational modulation strategies among the tested ligands. Notably, MMRi71 emerged as the most promising interface inhibitor, effectively locking the heterodimer into a highly stable and rigid conformation as evidenced by the lowest root mean square flexibility (RMSF = 1.03 Å) and a highly compact radius of gyration (RoG = 14.95 Å). This significant suppression of natural interface flexibility directly satisfies the study’s rationale of preventing the dynamic structural shifts essential for functional MDM2-MDMX crosstalk. Similarly, MMRi64 demonstrated strong stabilizing effects, maintaining low structural deviation (RMSD = 2.28 Å) and diminished flexibility (RMSF = 1.30 Å) relative to the apo protein. In contrast, the highlighted HLI373-bound system exhibited pronounced structural perturbation, yielding the highest deviation (RMSD = 5.11 Å) and hyper-flexibility (RMSF = 1.81 Å), indicating a disruptive mechanism rather than a rigidifying lock. Ultimately, these findings underscore the necessity of dynamic simulation frameworks in drug discovery and provide a clear computational rationale for prioritizing MMRi71 and MMRi64 as scaffold leads in developing potent therapeutics targeting the MDM2-MDMX oncogenic interface.

## 6. Future Perspective

There is a scarcity of solved, high-resolution co-crystal structures (i.e., lack of structural data) for small molecules bound specifically to the RING–RING interface. This makes homology modeling and rigid-ligand docking challenging. Therefore, obtaining co-crystal structures for small molecules bound specifically to the RING–RING interface should be a focus for anticancer drug design. Moreover, the RING heterodimer interface contains extensive hydrophilic contacts alongside hydrophobic clefts (e.g., Tyr489 residues). Current non-peptidic scaffolds struggle to achieve optimal electrostatic complementarity and binding free energy in this environment. Thus, it is critical to investigate the parameters in hydrophilic vs. hydrophobic balanced environments. In addition, the RING domains possess intrinsic oncogenic properties independent of p53, which is poorly accounted for in current screening protocols. Thus, this gap could be targeted by investigating the roles of protein-independent functions. Finally, ensemble-based virtual screening and homology modeling can be performed using AI-driven structural prediction tools to construct high-confidence models of the MDM2-MDMX RING heterodimer and implement ensemble docking with diverse compound libraries to account for structural flexibility.

## Figures and Tables

**Figure 1:**
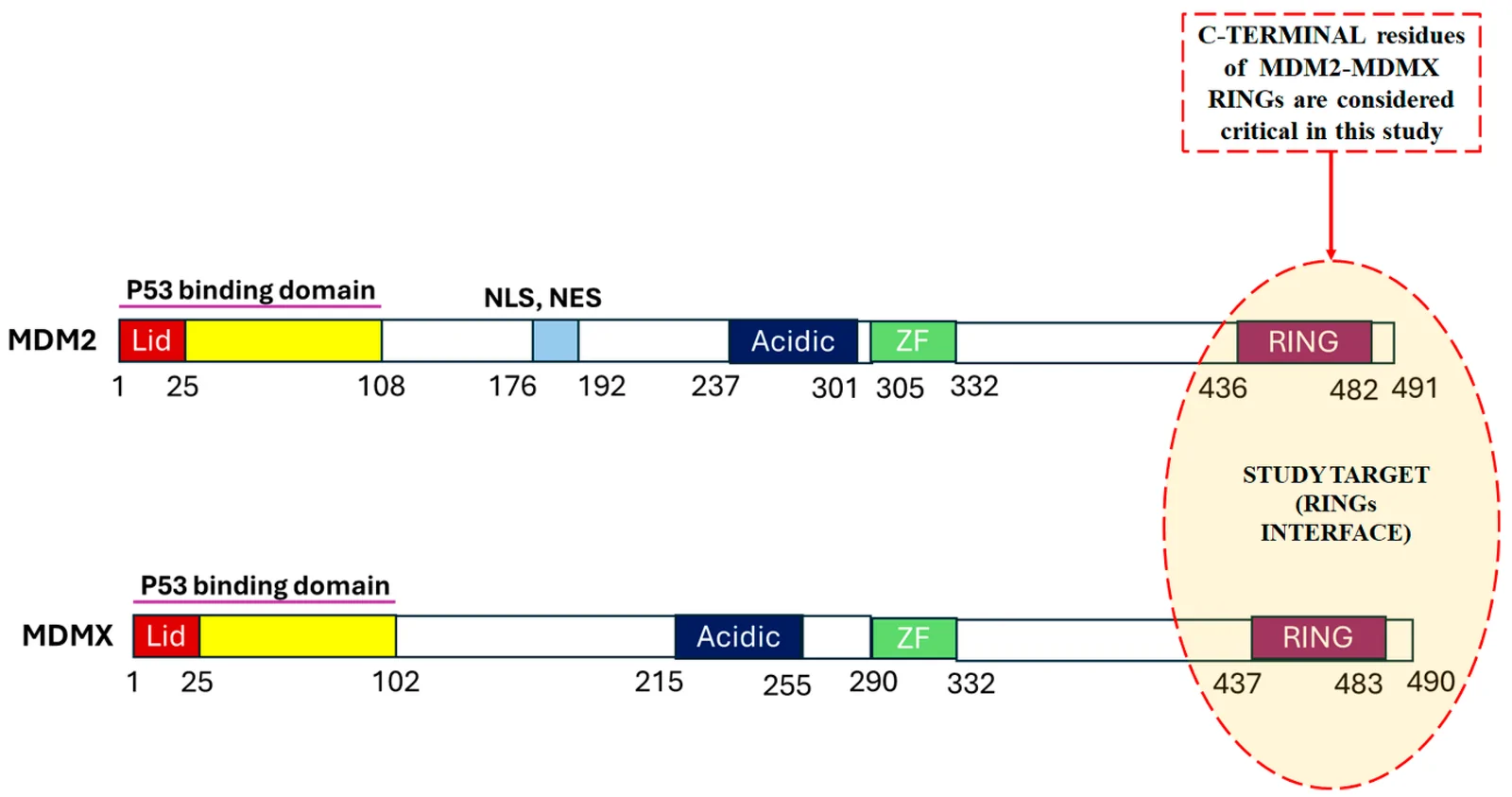
Schematic sections of MDM2 and MDMX proteins highlighting the amino acids within the RING domains.

**Figure 2:**
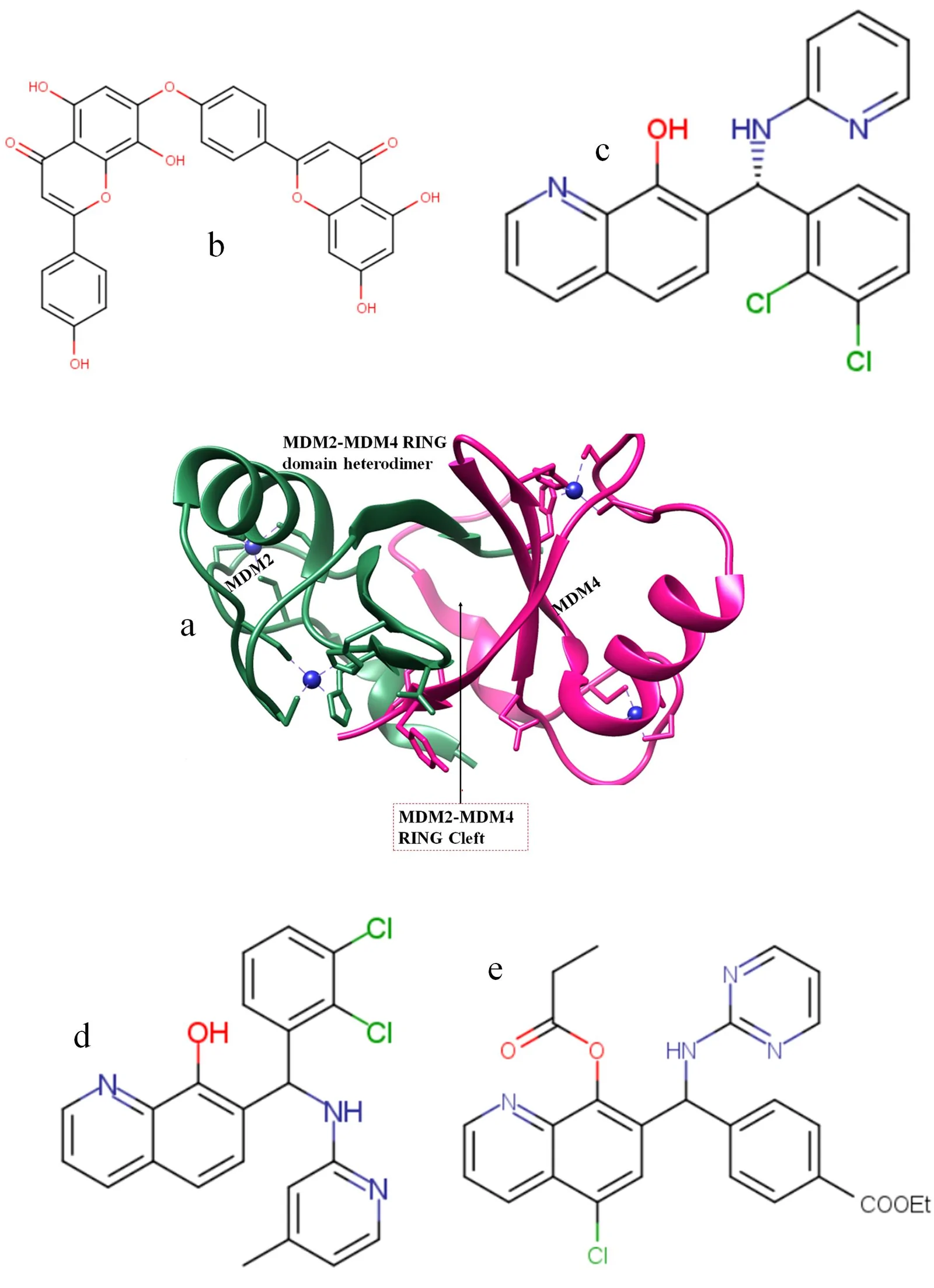
(**a**) The structure of the heterodimeric MDM2-MDMX RING domain, showing the interacting interface targeted as the binding pocket. The chemical structures of study inhibitors: Hinokiflavone, Hinok (**b**), *MMRi62* (**c**), *MMRi64* (**d**), and *MMRi71* (**e**).

**Figure 3:**
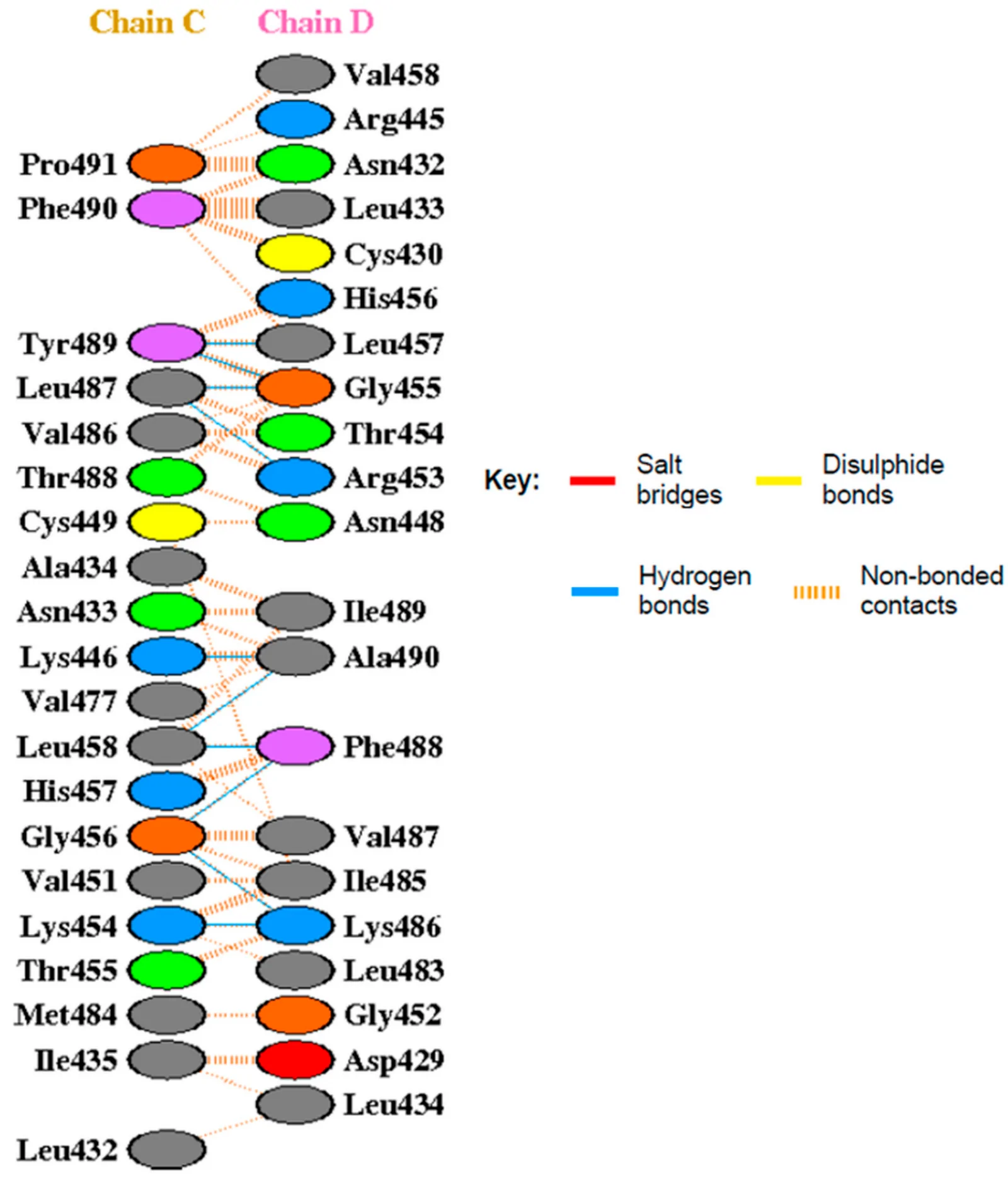
MDM2-MDMX RING interface residue mapping using *PDBSum* online open-source after the MDM2 RING protein–MDM2 RING protein docking using *ClusPro* open-source. Chain C residues are MDM2 RING, and Chain D residues are MDMX RING, showing the interacting forces, most importantly, hydrogen interactions.

**Figure 4:**
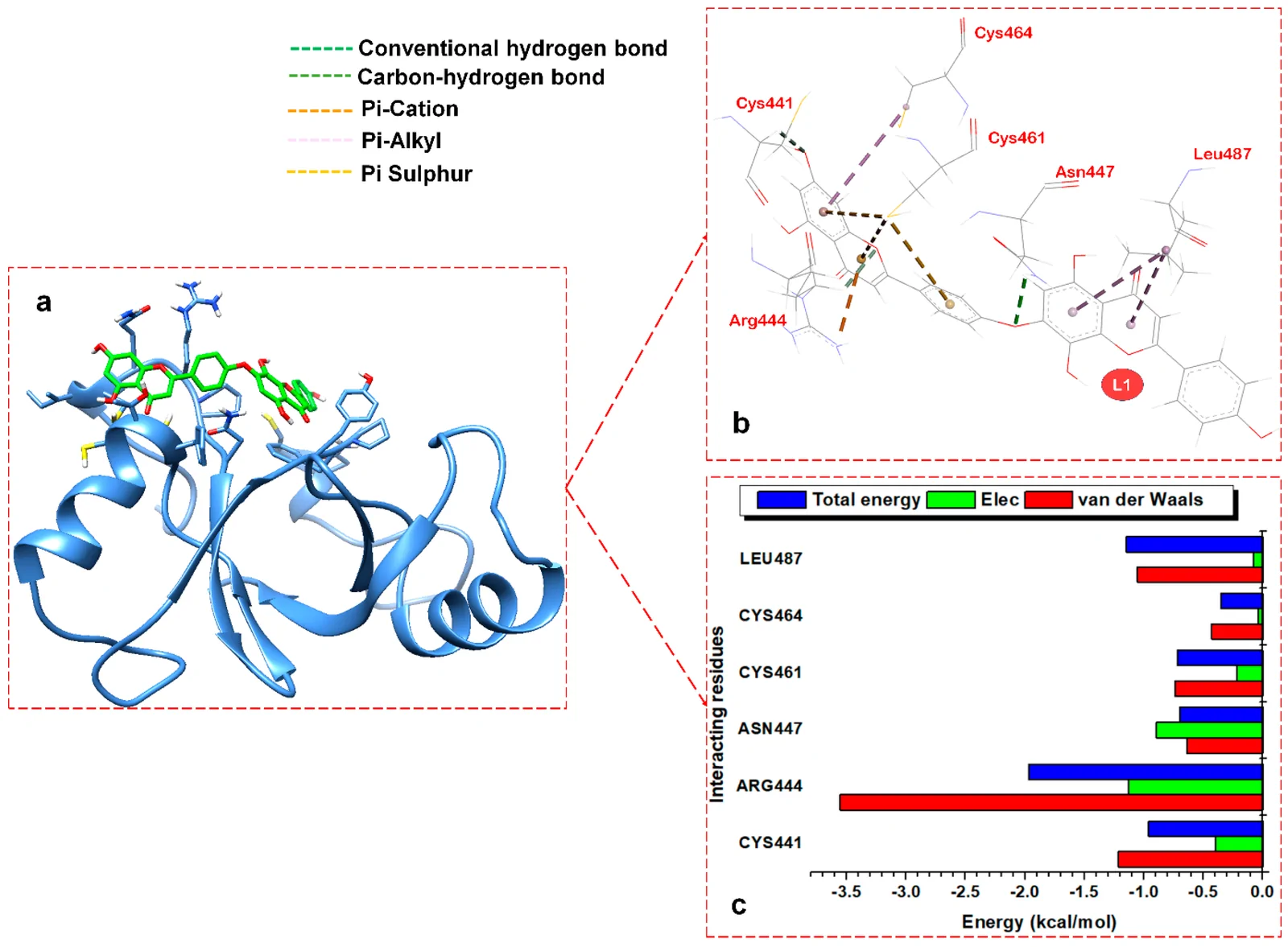
Surface visuals of L1/*Hinok* (cornflower color) complexed with (**a**) MDM2-MDMX (green), (**b**) 3D visual interaction network, and (**c**) per-residue energy contributions.

**Figure 5:**
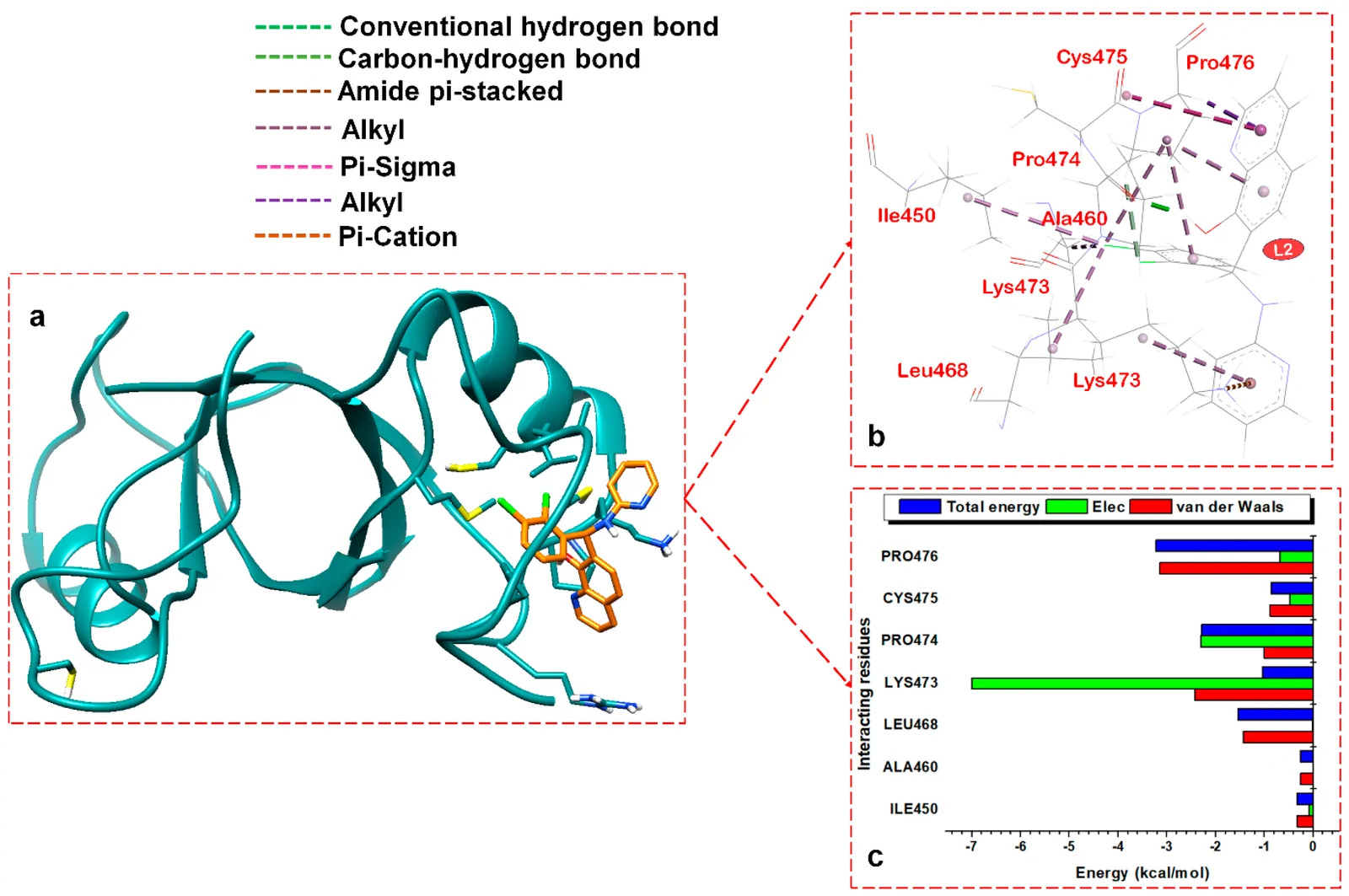
Surface visuals of L2/*MMRi62* (dark cyan) complexed with (**a**) MDM2-MDMX (orange), (**b**) 3D visual interaction network, and (**c**) per-residue energy contributions.

**Figure 6:**
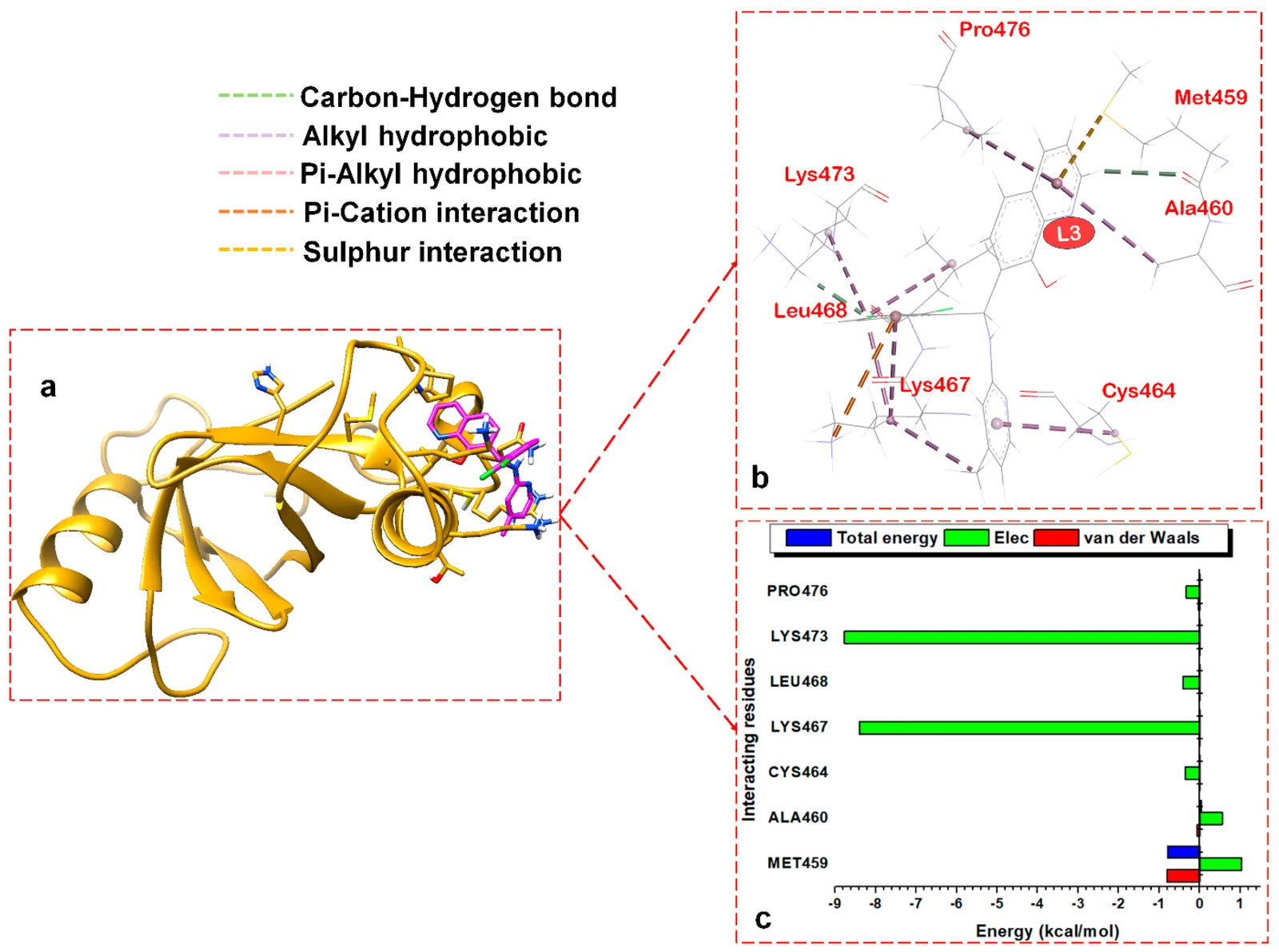
Surface visuals of L3/*MMRi64* (goldenrod) complexed with (**a**) MDM2-MDMX (magenta), (***b***) 3D visual interaction network, and (**c**) per-residue energy contributions.

**Figure 7:**
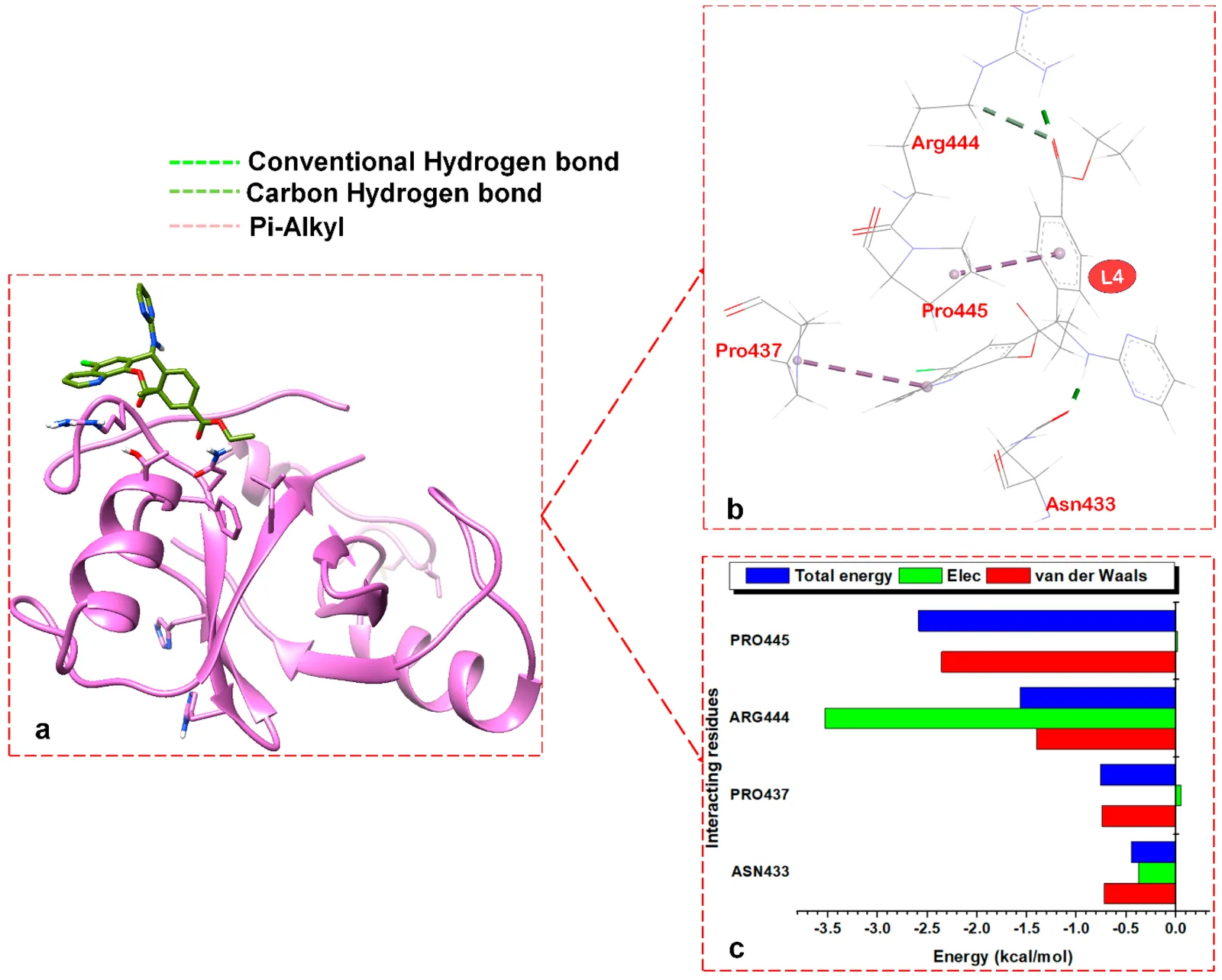
Surface visual representation of L4/*MMRi71* (orchid) complexed with (**a**) MDM2-MDMX (olive drab) protein, (**b**) 3D visual interaction network, and (**c**) per-residue energy contributions.

**Figure 8:**
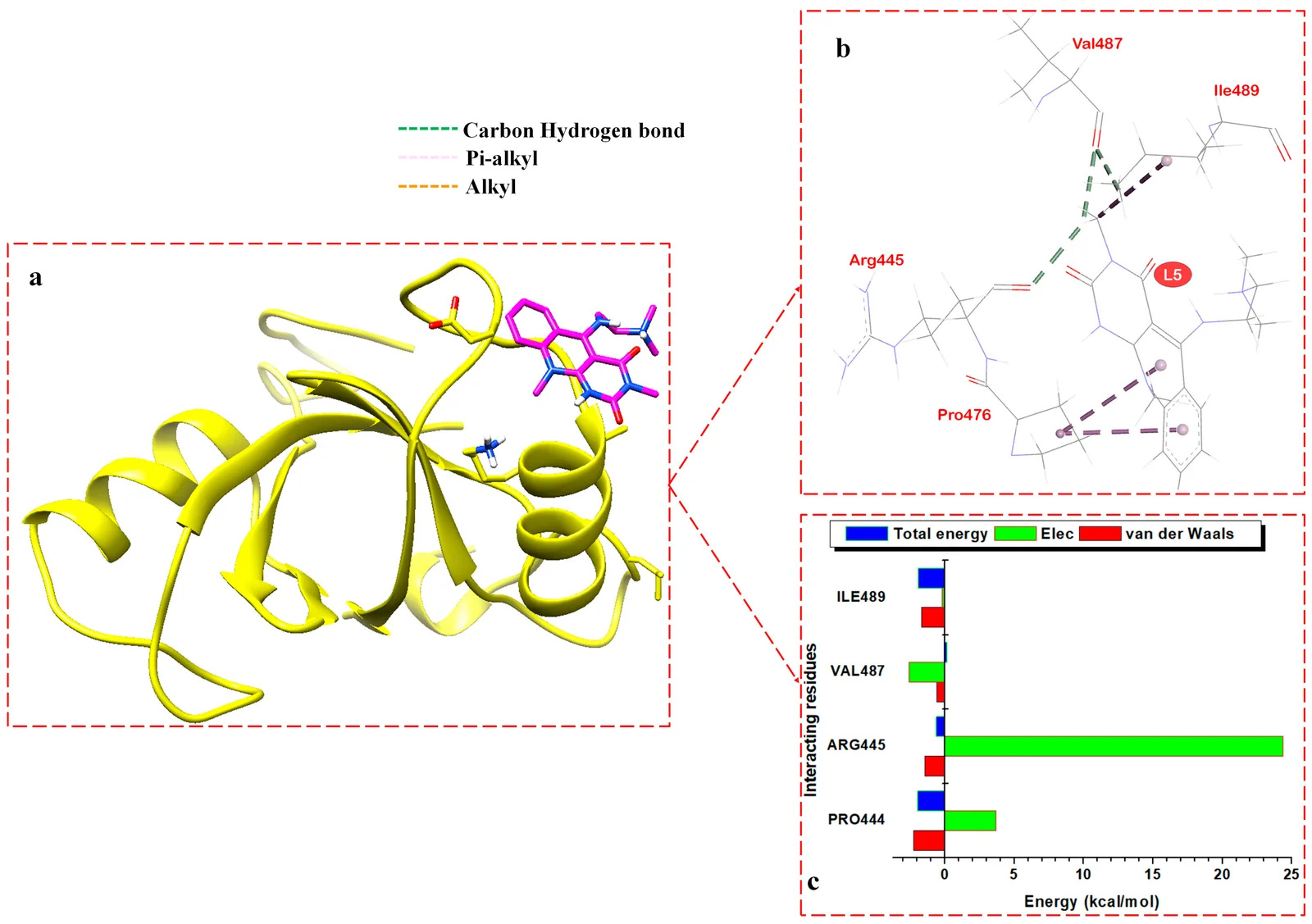
Surface visual representation of L5/*HLI373* (magenta) complexed with (**a**) MDM2-MDMX (yellow) protein, (**b**) 3D visual interaction network, and (**c**) per-residue energy contributions.

**Figure 9:**
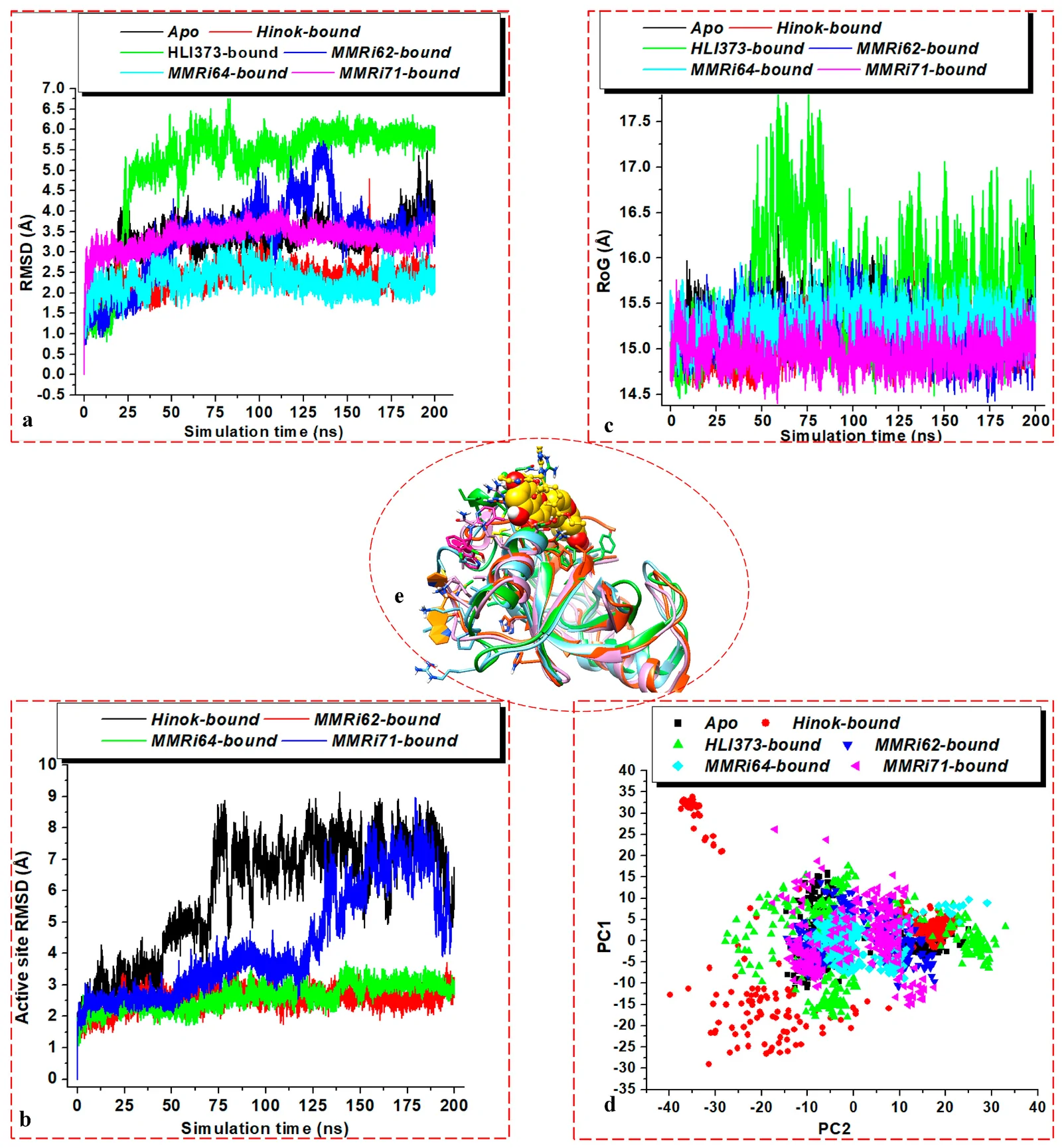
Comparative C-α atom plots of unbound and bound MDM2-MDMX RING domain, including RMSD (**a**); binding pocket RMSD (**b**); RoG (**c**); PCA (**d**), and (**e**) superimposed structures of the bound and unbound systems: Apo MDM2-MDMX RING (tan), *Hinok*-bound (dark red), *MMRi62*-bound (plum), *MMRi64* (green), and *MMRi71* (blue). Ligand color: *Hinok* (yellow sphere), *MMRi62* (deep pink ball and stick), *MMRi64* (magenta), and *MMRi71* (goldenrod, flat-shaped).

**Figure 10:**
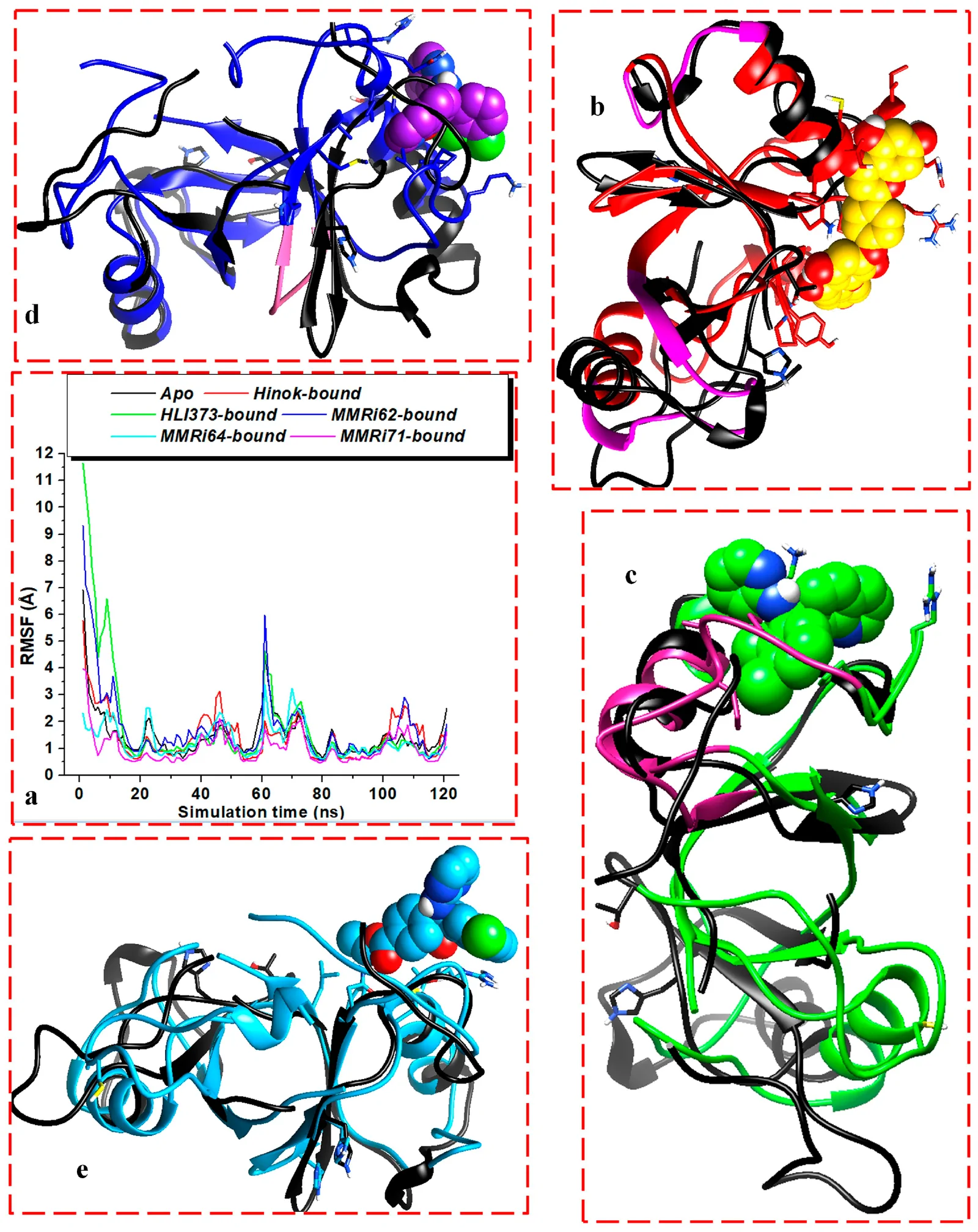
Each compound induced the protein differently. (**a**) C-α RMSF of the unbound and bound systems. (**b**) Hinok-bound complex (red), (**c**) *MMRi62*-bound (green), (**d**) *MMRi64*-bound complex (blue), (**e**) *MMRi71*-bound complex (cyan). The unbound MDM2-MDMX (black), Hinok (yellow ball and stick), MMR-62.

**Figure 11:**
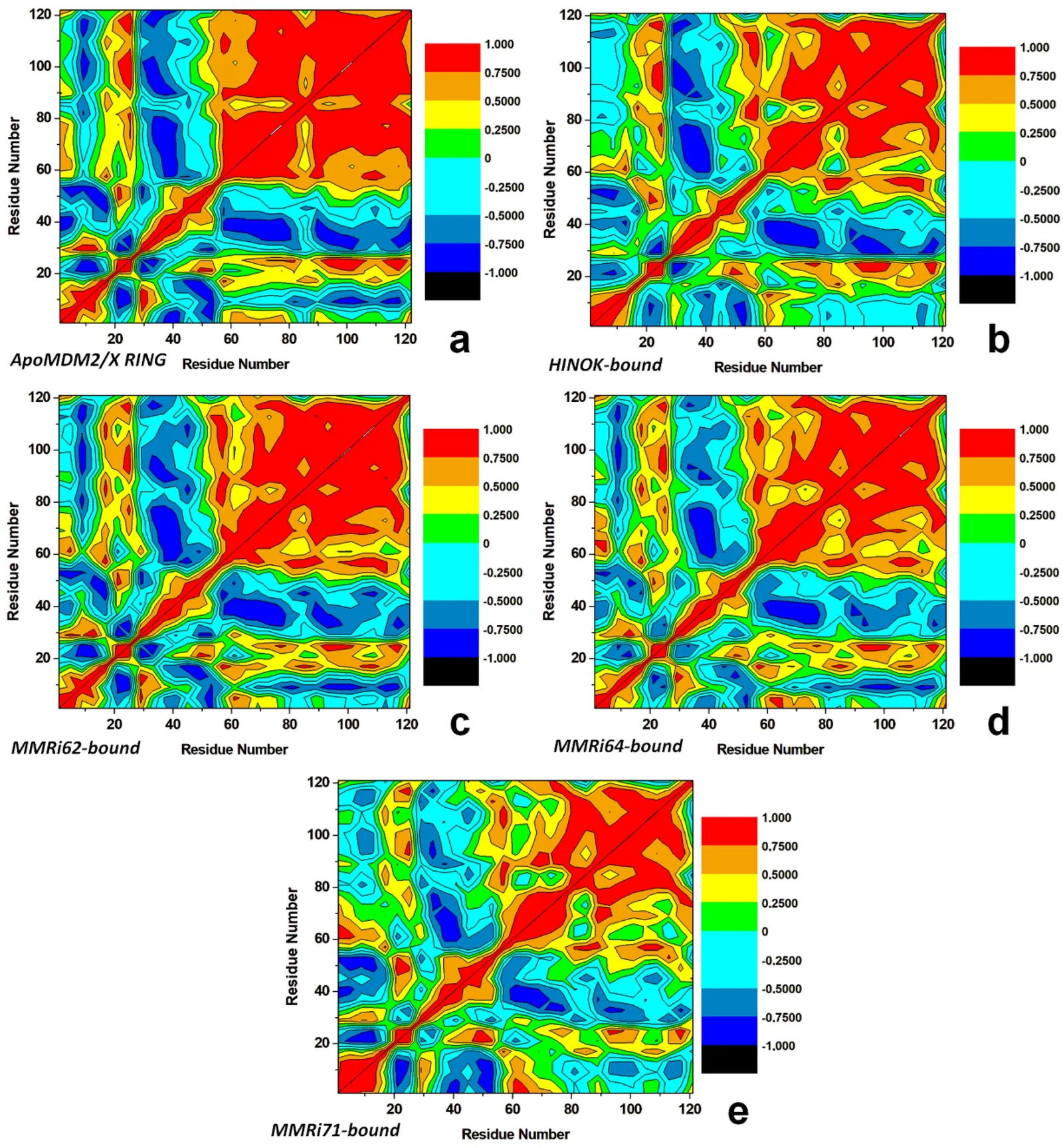
Correlation matrix plots of apo and ligand-bound systems; MDM2-MDMX RING (**a**) Apo, (**b**) Hinokiflavone-bound, (**c**) *MMRi62*-bound, (**d**) *MMRi64*, and (**e**) *MMRi71*.

**Figure 12:**
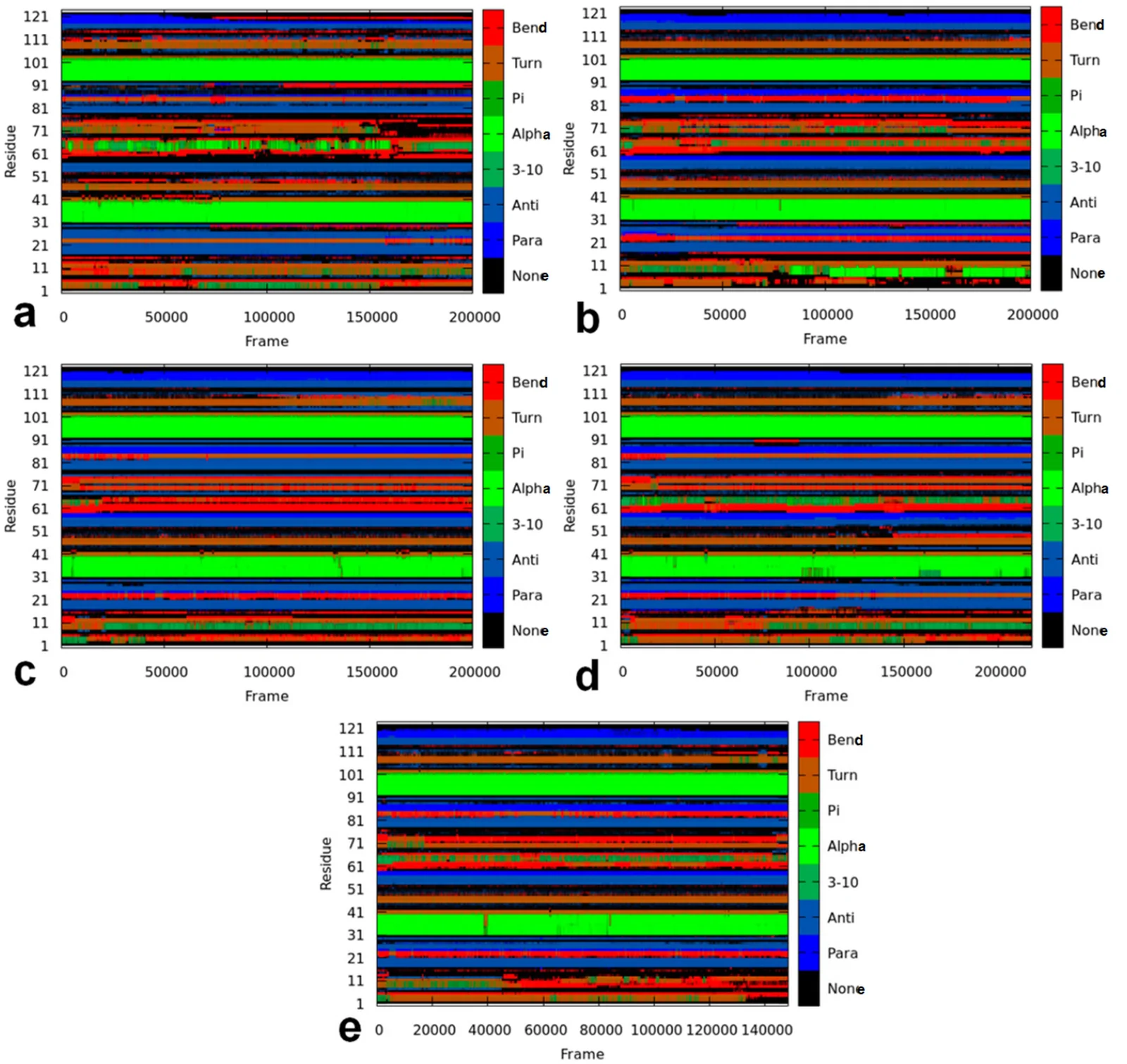
The DSSP charts for the MDM2-MDMX protein systems: Apo (**a**), *Hinok*-bound (**b**), *MMRi62*-bound (**c**), *MMRi64*-bound (**d**), and *MMRi71*-bound (**e**) complexes.

**Figure 13:**
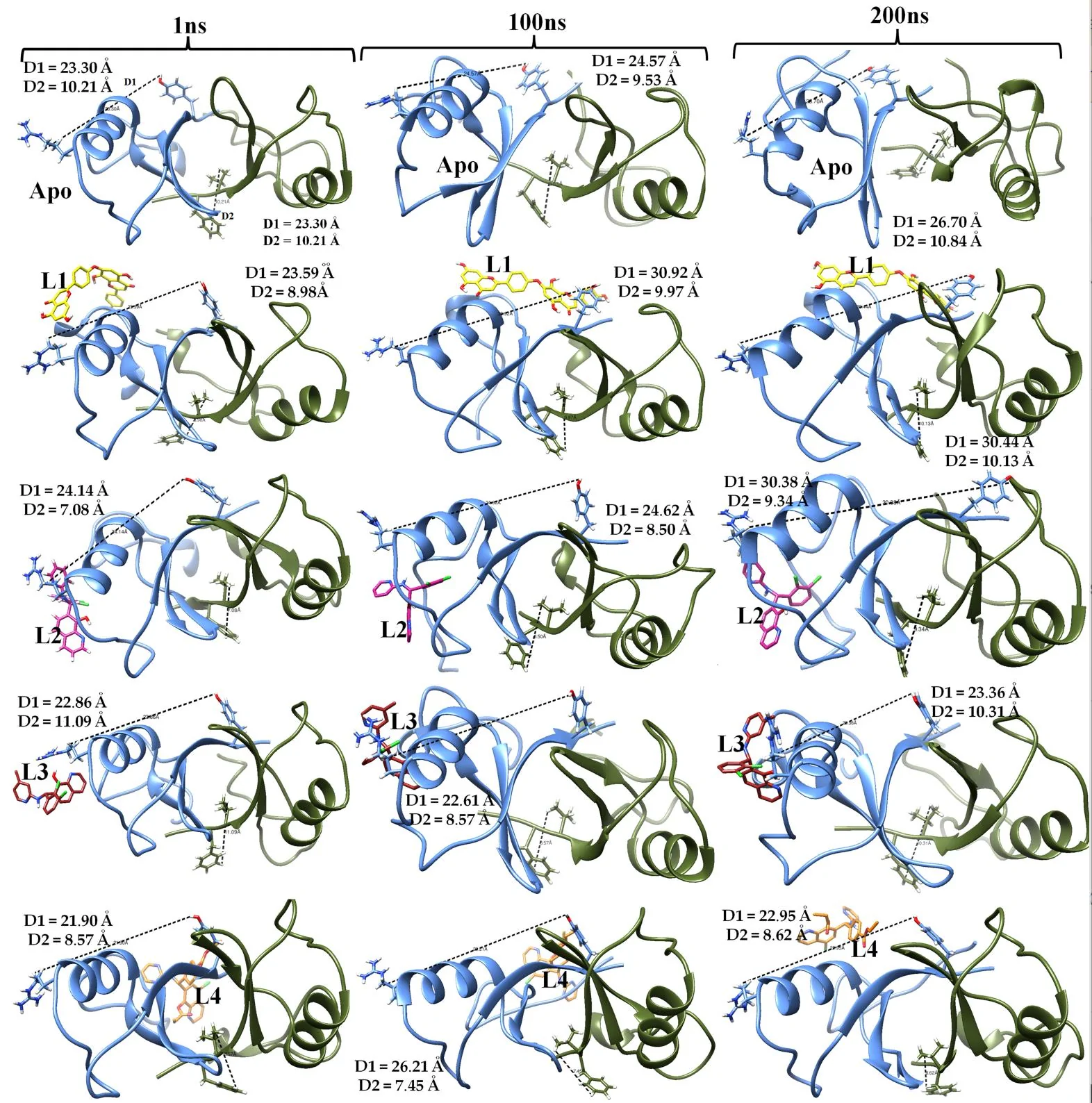
Showing distances between interacting amino acids Arg471-Tyr489 (D1) in MDM2 (cornflower blue) and Val487-Phe488 (D2) in MDMX/X for apo and ligand-bound MDM2-MDMX: *Hinok* (**L1**): yellow, *MMRi62* (**L2**): violet-red, *MMRi64* (**L3**): brown, *MMRi71* (**L4**): orange.

**Table 3:** The residues that interacted with each ligand during the MD simulation period.

Protein Complexes	Conventional Hydrogen	C-H Bond	Pi-Cation	Amide Pi-S	Pi-S	Pi-A/A
*Hinok*-bound	Asn447	Cys441 Arg444	Arg444 Cys461		Cys464	Leu487
*MMRi62*-bound	Pro474	Pro476	Lys473	Cys475		Ile450 Ala460 Leu468
*MMRi64*-bound		Met459 Lys473	Lys467		Met459	Ala460 Cys464 Leu468 Pro476
*MMRi71*-bound	Asn433 Arg444	Arg444				Pro437 Pro445
*HLI373*-bound		Arg445 Val487				Pro476 Ile489

**Table 4:** Characterizing the binding affinities of complexes–*Hinok*, *MMRi62*, *MMRi64*, *MMRi71* and *HLI373* bound to MDM2-MDMX RING computed using the MM/GBSA method.

	Energy Components (kcal mol^−1^)						
Complex	Δ*E_vdW_*	Δ*E_elec_*	Δ*G_gas_*	*E_GB_*	*E_SA_*	Δ*G_solv_*	Δ*G_bind_*
*Hinok*–bound	−32.55 (±5.43)	−14.24 (±8.38)	−46.79 (±8.63)	28.25 (±7.64)	−4.15 (±0.61)	24.10 (±7.70)	−22.69 (±5.05)
*MMRi62*–bound	−29.57 (±2.81)	−20.57 (±6.75)	−50.14 (±7.35)	27.65 (±5.61)	−3.92 (±0.37)	23.72 (±5.47)	−26.42 (±3.27)
*MMRi64*–bound	−22.71 (±5.18)	−15.97 (±8.30)	−38.69 (±10.86)	24.99 (±7.40)	−3.04 (±0.58)	21.95 (±7.25)	−16.73 (±6.27)
*MMRi71*–bound	−28.17 (±6.50)	−19.00 (±5.89)	−47.17 (±10.13)	30.42 (±5.98)	−3.55 (±0.81)	26.87 (±5.45)	−20.30 (±6.03)
*HLI373*–bound	−24.51 (±2.43)	717.86 (±29.67)	693.35 (±29.41)	−704.46 (±29.17)	−2.98 (±0.26)	−707.44 (±29.41)	−14.09 (±2.54)

*ΔE_elec_* (electrostatic), *ΔE_vdw_* (van der Waals), *ΔG_bind_* (calculated total free binding energy), *ΔG_gas_* (gas-phase energy), and *ΔG_solv_* (solvation free energy).

## Data Availability

The protein used in the experiment was retrieved from the RCSB Protein Data Bank (RCSB PDB) (https://www.rcsb.org/structure/2VJE). The 3D MMRi6 and *MMRi64* were retrieved from https://pubchem.ncbi.nlm.nih.gov/#query=MMRi64+and+MMRi6, the PubChem database. The removal of the unused protein chain, non-standard ligands, and required visualization during the study was performed using the UCSF Chimera software (https://www.cgl.ucsf.edu/chimera/). Other interactive visualization and analysis of molecular structures and related data were performed using BIOVIA DS Visualizer version 2021 (https://discover.3ds.com/discovery-studio-visualizer-download) and Molegro Molecular Viewer www.molegro.com. AutoDock Vina, used for the molecular docking studies, is freely available at https://doi.org/10.1021/acs.jcim.1c00203. The molecules were optimized using Avogadro’s Tool, which is made freely available at https://avogadro.cc/. The PMEMD CUDA GPU version provided with the AMBER package, available at the Centre for High-performance Computing (CHPC) in Cape Town, South Africa, was used to run the MD simulations (https://www.chpc.ac.za/) and (https://users.chpc.ac.za/). All the parameters used for the calculations in the MD simulation are provided in detail in the “Methodology” section. All data, including docking results, docking poses, MD simulation trajectories, RMSD, RMSF, RoG, PCA, PRED, MMGBSA, DCCA, Snapshots, and others, were made available at https://github.com/Jesutomisin0707/Analysis-of-the-Mouse-Double-Minute-RING-Interface-project, https://github.com/Jesutomisin0707/open-source-code-for-running-AMBER-MD-simulations, and the rest of the data are available at https://github.com/Jesutomisin0707/Analysis-of-the-Mouse-Double-Minute-RING-Interface-project-second_200-ns-MD_simulations, all accessed on 6 August 2025.

## Supplementary Materials

The following supporting information can be downloaded at: https://www.mdpi.com/article/10.3390/biomedicines14102366/s1, Supplementary Figure S1. Molecular docking interaction networks between the active site MDM2/MDMX RING and each inhibitor, including *HLI*373, *MMRi*62, *Hinok*, *MMRi*64, and *MMRi*71.

## Abbreviations

The following abbreviations are used in this manuscript:

MDM2	Mouse double minute 2
MDM4/X	Mouse double minute 4
RMSD	Root mean square deviation
RMSF	Root mean square fluctuation
RoG	Radius of gyration
PCA	Principle component analysis
DCCM	Dynamic cross-correlation matrix
DSSP	Define secondary structure of a protein
C-α	Backbone alpha-carbon atoms
