# Supplementary material for: Computational Analysis of the Homologous Mouse Double RINGs Interface for Targeted Anticancer Drug Design
Source: Biomedicines. 2026 Oct 17;14(10):2366. doi: 10.3390/biomedicines14102366 (PMC13701783; doi:10.3390/biomedicines14102366)
Supplement: Supplementary file 1 [file biomedicines-14-02366-s001.zip › biomedicines-4533120-supplementary.pdf]

Supplementary Materials

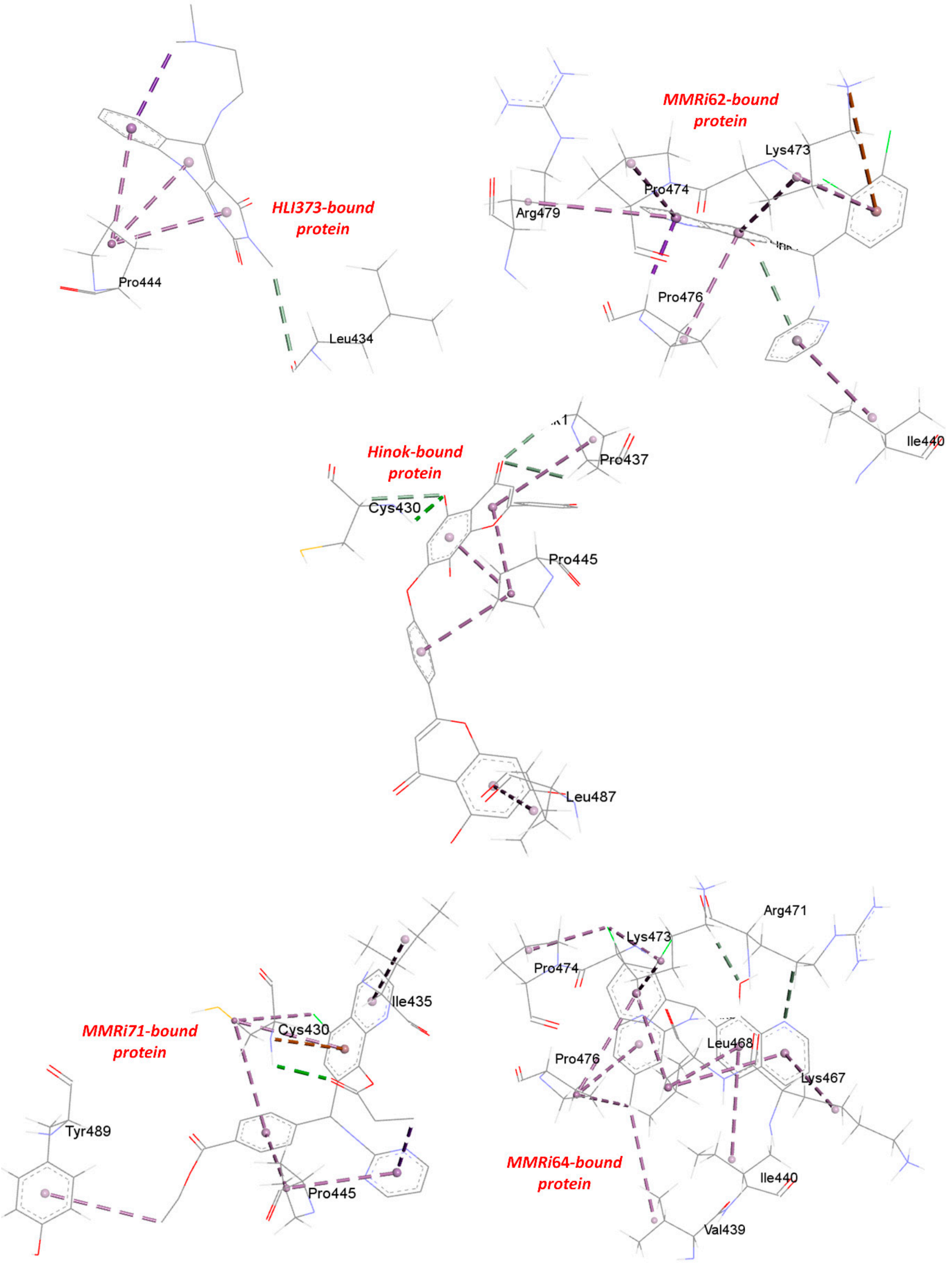

Supplementary Figure S1. Molecular docking interaction networks between the active site MDM2/MDMX and each inhibitor, including *HLI*373, *MMRi*62, *Hinok*, *MMRi*64, and *MMRi*71.
